# Chicken cecal microbial functional gene content and resistome differ by age and barn disinfection practice

**DOI:** 10.1128/spectrum.03737-25

**Published:** 2025-12-26

**Authors:** Yi Fan, Tingting Ju, Tulika Bhardwaj, Douglas R. Korver, Benjamin P. Willing

**Affiliations:** 1 Department of Agricultural, Food and Nutritional Science, Faculty of Agricultural, Life and Environmental Sciences, University of Alberta 3158, Edmonton, Alberta, Canada; 2 Department of Animal Sciences, Purdue University 311308 , West Lafayette, Indiana, USA; Cleveland Clinic Lerner Research Institute, Cleveland, Ohio, USA

**Keywords:** functional gene content, antibiotic resistance genes, poultry gut microbiome, chemical disinfectants

## Abstract

Chemical disinfectants and water-wash methods are widely employed in sanitizing broiler chicken barns. Studies showed that disinfectants affect environmental microbial composition and antibiotic resistance genes (ARGs). However, little is known regarding how barn disinfection treatments impact the chicken gut resistome and microbial functional gene content. The current study compared the effects of disinfection and water-wash method on the gut microbiome and resistome of commercial broilers using a crossover experimental design after two production cycles at seven barns. Shotgun metagenomic sequencing performed on cecal contents collected at days 7 and 30 also allowed the evaluation of age-associated characteristics of the microbiome. The age of the chickens had the largest effects on the resistome, with younger birds having higher relative abundance of total ARGs (*P* < 0.05) and differences in resistance mechanism; however, functional gene content and resistome differences were also identified by barn sanitation practice. At day 7, chickens in chemically disinfected barns had decreased gene content related to amino acid synthesis compared to the water-wash group. Additionally, genes related to stringent response were enriched in chickens raised under chemically disinfected conditions (FDR-P < 0.05), suggesting the selection for stress resistance. Lower abundance of genetic pathways encoding amino acid biosynthesis associated with cecal *Helicobacter pullorum* was observed in the disinfection group at day 30 compared to the water-wash group, with the same pattern in short-chain fatty acid biosynthesis (FDR-P < 0.05). Overall, while the use of disinfectants in barn sanitation slightly affected the relative abundance of some ARGs in the gut, age had a dominant effect on the microbial gene function and resistome.

**IMPORTANCE:**

This is the first study to evaluate the effect of sanitation practices on microbial functional gene content and resistome of chickens in a commercial setting. It is also amongst the biggest metagenomics studies on the gut microbiome of broiler chickens. It provides new insights into the changes in resistance profiles with age that agree with other studies examining maturation of the microbiome in other species. Finally, the current study provides valuable insights for informing industry sanitation practices and future studies on broiler gut microbiome and resistome.

## INTRODUCTION

Livestock farming accounts for over 50% of antibiotic usage globally (1). Compared with other livestock species, chickens were reported to have the highest density of antibiotic resistance genes (ARGs) due to the high stocking density and short production cycle (2). High ARG abundance in chicken production can lead to reduced sensitivity to antibiotic treatments on infectious disease. Furthermore, it also increased the risk of ARG transmission, often using bacteria as a vessel, to humans through the food chain or environmental routes. The transmission of ARGs to human-associated bacteria can complicate the treatment of human infections, contributing to the global antimicrobial resistance crisis, with an estimated 1.27 million deaths directly attributed to antibiotic resistance in 2019 (3). Currently, to reduce bacterial load in poultry farming, biocidal agents such as benzalkonium chloride (BAC), hydrogen peroxide, glutaraldehyde, ethanol, and sodium hypochlorite have been widely applied for facility disinfection purposes (4). While chemical disinfectants can inhibit antibiotic-resistant bacteria and destruct ARGs through oxidation, they may also induce bacterial adaptation, potentially through promoting antibiotic resistance through co-selection (5). The impact of chemical disinfectants on ARG proliferation remains uncertain, with some studies suggesting that they may act as stressors, stimulating the proliferation and transfer of microbial ARGs (6–8). For example, BAC, a commonly used quaternary ammonium compound, has been associated with increased resistance to ampicillin, cefotaxime, and sulfamethoxazole in various food-related bacterial isolates (6, 9), along with co-selected ARGs (10). Some studies, on the other hand, suggested that chemical disinfectants may contribute to controlling antibiotic resistance by reducing the abundance of ARGs. For instance, quaternary ammonium compounds and sodium hypochlorite used in treating swine manure have been reported to decrease the abundance of selected ARGs [*erm(B), erm(C), erm(F), intI1, tet(Q),* and *tet(X*)] (11). Oxidants like chlorine and hydroxyl radicals have also demonstrated potential in eradicating ARGs presented in both *E. coli* cells and plasmids (12). With the controversial effect of chemical disinfectants on ARGs, there is limited information available regarding the effects of chemical disinfectant-treated rearing environments on the resistome in the gut of animals. In this sense, barn cleaning practices, which involve chemical disinfectants, may impact the presence and persistence of ARGs throughout production cycles. Consequently, from the perspective of food safety and environmental sustainability, it is important to explore the influence of chemical disinfectant usage in barn cleaning.

In addition to environmental factors like disinfection practices, the age of chickens plays a crucial role in the development of the gut microbiota. Studies have shown that the chicken gut microbiome undergoes successional changes as they mature (13, 14). The microbial abundance of the microbiome increases during the first week of life, with compositional shifts of important taxa occurring over several weeks (15). Understanding these age-dependent microbiome dynamics is essential, as they can play an important role in shaping the microbial functional gene content and the overall ARG profile within the gut.

In broiler chicken production, barn sanitation has been used with the goal of enhancing biosecurity and preventing disease transmission between flocks. Both full sanitation with chemical disinfectants (FD) and water-wash (WW) method are widely employed in the Canadian poultry production system (16). To date, large-scale metagenomic studies have advanced understanding of the poultry gut resistomes by characterizing the ARG carriage, relative abundance, and diversity of ARGs in the gut of commercial broiler chickens (17–23). These works have elucidated important dynamics of ARG profiles linked to factors such as different production modes (21, 22), antibiotic administrations (20, 22, 23), geographic location (17, 18), and bacterial community composition (19). However, despite these valuable insights, the present work represents the first research to directly assess how chemical disinfectants applied for barn sanitation impact the chicken gut resistome at commercial production scale. Our previous study showed that FD resulted in an undesired increased carriage of *Campylobacter jejuni* accompanied by alterations in the cecal microbial composition, revealed by 16S rRNA gene amplicon sequencing (24). Full disinfection also resulted in lower cecal short-chain fatty acids (SCFAs) when compared to the WW (24). Additionally, as age has been identified as an influencing factor in the gut microbiota composition, understanding how the successional changes in microbiota affect the microbial functional gene content and the ARG profile is essential for a comprehensive understanding of the chicken gut microbiome. In the current study, we sought to gain greater insight into the effects of barn sanitation and age on the functional gene content of the gut microbiome, specifically in terms of microbial metabolic capacity and the profile of antibiotic resistance genes.

## MATERIALS AND METHODS

### Animals

The current study was performed according to the guidelines of the Canadian Council on Animal Care with approval of the University of Alberta Animal Care and Use Committee (AUP00002377). Broiler chicken management and barn cleaning practices were described previously (24). Briefly, the animal study was conducted on seven commercial broiler chicken barns owned and managed by the same producer in Alberta, Canada, between the months of June to September. All barns were similarly engineered single-story production houses with cement floors (Fig. S1). During each production cycle, samples were collected from barns that had undergone two consecutive rounds of repeated cleaning treatments, including both FD and WW. For FD treatment, manure and litter were completely removed from the barn after chickens were depopulated. Subsequently, chemical disinfection was performed using foam containing 7% sodium hydroxide, 7% 2-(2-2-butoxyethoxy) ethanol, 6% sodium laureth sulfate, 5% sodium N-lauroyl sarcosinate, and 5% tetrasodium ethylenediaminetetraacetic acid on all surfaces within the facilities, followed by high- and low-pressure water rinse with water temperature set at 35℃. After the facilities were air-dried, foam containing 10% glutaraldehyde, 10% benzalkonium chloride, and 5% formic acid was applied to surfaces of the facilities for 60 min, followed by high-pressure water rinse, overnight air-dry, and fresh litter placement. For WW treatment, manure and used litter were removed, followed by low-pressure water rinse with the water temperature set at 35°C for all facility surfaces, air-dry overnight, and fresh litter placement (wood shaving, 10–15 cm deep).

A cross-over design was performed, which resulted in a total of 14 production flocks with seven flocks assigning to each treatment to ensure that every barn had gone through both FD and WW treatments. The study included a total of 140 chickens with 35 chickens sampled from each treatment at day 7 (D7) and day 30 (D30) to analyze their cecal microbiota. Sampling at D7 was intended to represent the starter phase, whereas sampling at D30 was chosen to be as close as possible to slaughter (day 32) while avoiding the fasting period prior to processing. In each production flock, Ross 308 broiler chicks were placed within 12 h post-hatch and confined to half of the house, then allowed access to the entire house starting at D7. The flock size was approximately 14,000 birds, with a final stocking density of 30 kg/m^2^. All chickens were fed the same diet without antibiotics *ad libitum* and sent for processing at 32–35 days of age when the average target live weight of 1.8 kg was reached. At D7 and D30 of age, five broilers per flock were randomly selected from five different areas within each barn (Fig. S1) and euthanized using cervical dislocation. Approximately 300 mg of cecal contents were collected in autoclaved 1.5 mL Fisherbrand Microcentrifuge tubes (catalog # 05-408-129) using sterile technique, placed on dry ice until being transported to the lab, and stored at −80°C.

### Shotgun metagenomic sequencing

The total DNA extraction process was conducted as previously outlined (24). In summary, DNA extraction was carried out from homogenized cecal contents using the QIAamp Fast DNA Stool Mini Kit (Qiagen, Valencia, CA, USA), including an extra bead-beating step utilizing approximately 200 mg of garnet beads at a speed of 6.0 m/s for 60 s (FastPrep-24 5G instrument; MP Biomedicals Inc., Santa Ana, CA, USA). DNA samples were quantified using a determined by using a Quant-iT PicoGreen dsDNA assay kit (Invitrogen, Waltham, MA) and assessed for purity using a Nanodrop 2000c spectrophotometer (Thermo Fisher Scientific). For each sample, approximately 300 ng of extracted DNA meeting purity criteria (OD 260/280 ratio of 1.8, OD 260/230 ratio between 2.0 and 2.2, and concentration between 20 and 100 ng/μL) was sent to Genome Quebec Innovation Centre for shotgun metagenomic sequencing. Library preparation and shotgun sequencing were performed by the Genome Quebec Innovation Centre (Montreal, Canada). Prior to library preparation, the Genome Quebec Innovation Centre performed an additional quality control step, reassessing the DNA quantity using their own Qubit instrument to ensure having at least 150 ng of DNA for successful library construction. Libraries for all samples including the ZymoBIOMICS Gut Microbiome Standard (cat. # D6331, Zymo Research Corp. CA, USA) were prepared using the Nextera DNA Flex Library Prep Kit (Illumina Inc., San Diego, CA, USA), and the subsequent shotgun sequencing was executed on a NovaSeq 6000 system (Illumina Inc., San Diego, CA, USA). Read quality was assessed using FastP v0.23.2, and low-quality reads, sliding windows, adaptors, polyG, and duplicated sequences were removed (25). Kneaddata v0.10.0 was used to remove host DNA contaminants (https://github.com/biobakery/kneaddata). Briefly, a chicken host reference database was built using bowtie2 v2.4.1 with genome *Gallus_gallus* 105 release from Ensembl (26). Subsequently, reads aligned to the host genome were removed as host contaminants. Microbial taxonomic classification was profiled using Kraken2 (v2.1.2) (27), and the relative abundance estimation was conducted by Bracken2 (v2.6) (28). Bacterial taxa that appeared in less than 5% of the samples were filtered out for subsequent analyses. Genome assembly was performed via megahit (v1.2.9) with default parameters (29). The abundance of functional genes and enriched pathways was estimated using HuMAnN3 (v3.0.1) based on the UniProt 90 database followed by annotation using the Metacyc database (30, 31). The relative abundance of aligned genes and pathways was normalized to copy numbers per million reads using the HuMAnN3 utility scripts (31).

Antibiotic resistance-encoding genes were annotated against the Comprehensive Antibiotic Resistance Database (CARD, version 3.1.4) via Resistance Gene Identifier (RGI, version 5.1.0) with a cutoff set at 95% identity (32). To reveal the distributions of microbial taxa, functional genes, and ARGs across samples, principal coordinate analysis (PCoA) based on Bray-Curtis distance metric was performed via vegan package (1.11-0) in R (version 3.6.1). To evaluate dispersion, the “betadisper” function in the vegan package (1.11-0) was used to calculate distance to centroid. Due to potential biases introduced by variations in the relative abundance of bacterial taxa, 16S rRNA copy numbers harbored by different bacterial species as well as fluctuation caused by ARGs located on mobile genetic elements, identified ARGs per sample were normalized to reads per million total ARG reads for subsequent comparisons.

### Statistical analyses

Permutational multivariate analysis of variance (PERMANOVA) tests were used to determine clustering significance using the adonis function in the vegan package (1.11-0) in R (version 3.6.1, false discovery rate (FDR) adjusted *P* < 0.05), setting housing as a blocking effect. Differentially abundant microbial taxa, gene pathways, and ARGs associating with treatments or age were identified using LDA effect size (LEfSe) implemented in the lefser R package (Bioconductor version 3.15) with housing as “blockCol” (blocking effect). A significant cut-off was set at LDA score > 2 and FDR-adjusted *P* < 0.05. Differential abundance analysis of profiled gene pathways and ARGs between groups was performed using the DESeq2 package in R (Bioconductor version 3.15) (33). Significance of differential abundance was determined with FDR-adjusted *P* < 0.05 and log2 fold change > 1. Subsequently, the effect of housing factor was assessed by the likelihood ratio test in DESeq2 with an adjusted *P*-value threshold of 0.1. Except for the statistical analyses mentioned above, GraphPad Prism 8 (Graphpad Software, San Diego, CA, USA) was used to conduct statistical analyses. To determine differential significance of the microbiome alpha diversity indices, the Kruskal–Wallis test was used with significance set at *P* < 0.05. Two-way ANOVA was used to assess total ARG reads between different barn sanitation practices and sampling time points. The Spearman correlation was used to correlate ARG abundance and the relative abundance of bacterial species. Correlation significance was determined by the corr.test function with FDR-adjusted *P* < 0.05, and a moderate association was determined by ∣R∣ > 0.4. Correlation was visualized using the corrplot package in R (version 3.6.1).

## RESULTS

### Cecal microbial structures and functional capacities were impacted by both barn cleaning methods and age

**Fig 1:**
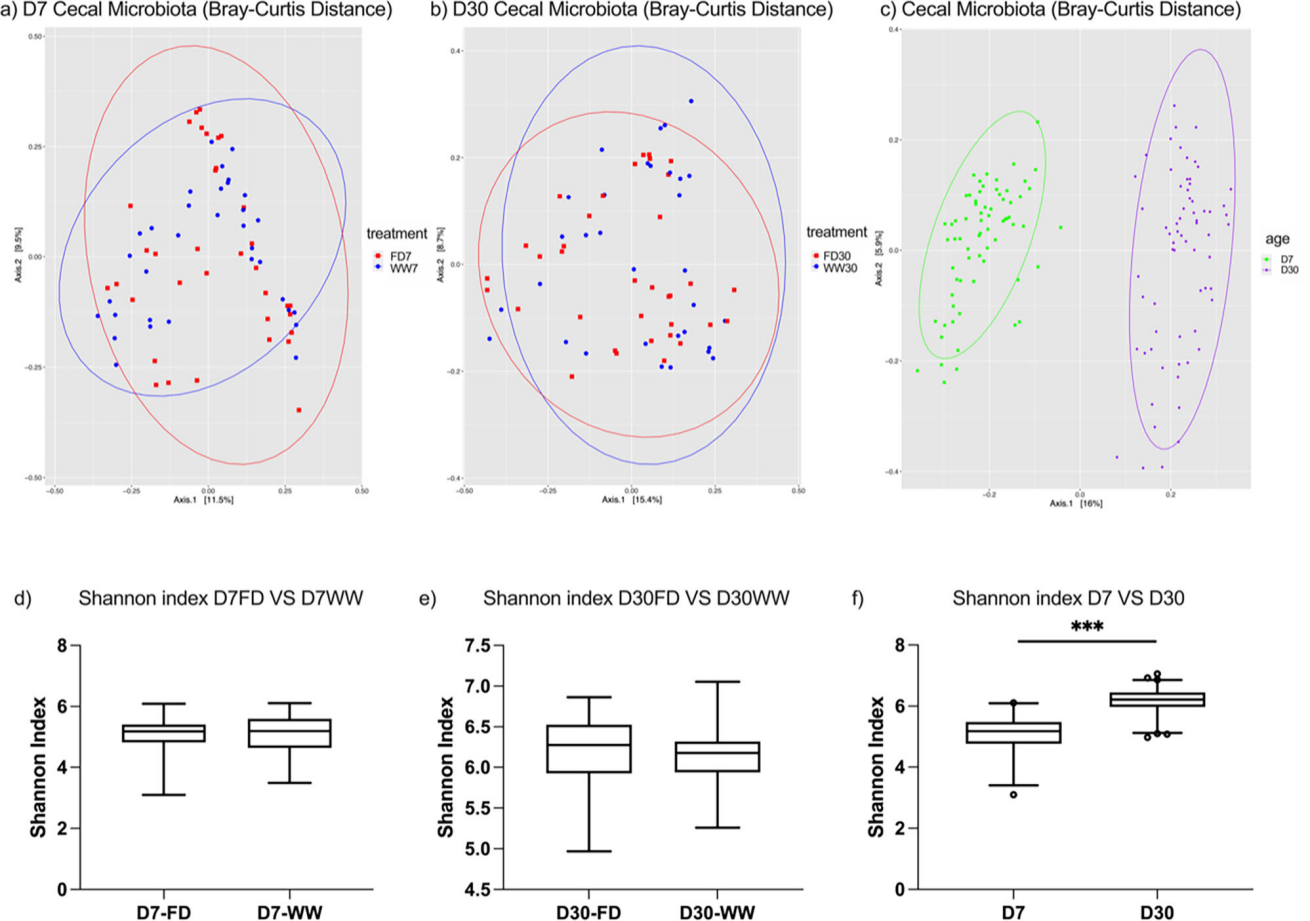
Broiler chicken cecal microbial structure affected by the barn cleaning practices and age. (**a–c**) Factors impacting the cecal microbial structures. Age had a major impact on microbial compositions, whereas the cleaning methods had a modest effect on the D30 cecal microbiota. (**d–f**) Factors affecting the cecal microbial alpha-diversity as indicated by the Shannon index. At D30, the richness and evenness of the cecal microbial species significantly increased compared to that at D7. FD, full disinfection; WW, water-wash; ***, *P* < 0.001.

**Fig 2:**
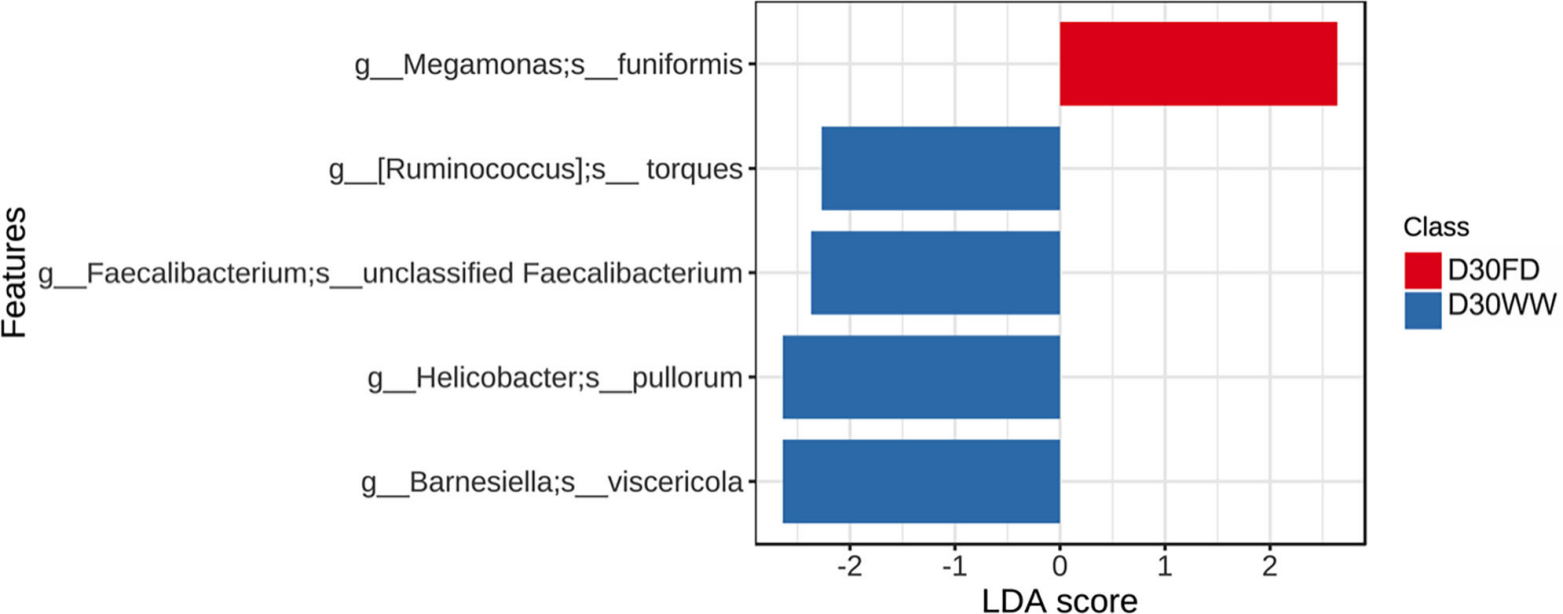
Differentially abundant bacterial species between barn cleaning practices at day 30 suggested by LEfSe analysis (LDA score > 2 and FDR-adjusted *P* < 0.05). At day 30, *Ruminococcus torques, Barnesiella viscericola, Helicobacter pullorum*, and *Faecalibacterium prausnitzii* were more abundant in the ceca of the chickens from the WW treatment group, whereas *Megamonas funiformis* was more abundant in the chicken cecal microbiota of the FD group. FD, full disinfection; WW, water-wash.

In this study, shotgun metagenomic sequencing was used to provide a comprehensive profile of the cecal microbial community to further assess the effects of barn cleaning methods, yielding an average read depth of 52,133,725.06 ± 1,265,988.36 quality-controlled reads per sample (mean ± SEM) for downstream analyses. These reads were processed using Kraken2, resulting in 34,252,401.09 ± 6,541,062.17 reads per sample aligning to the RefSeq bacteria database. At the phylum level, Firmicutes, Bacteroidetes, Proteobacteria, and Actinobacteria made up the majority cecal microbial communities both at days 7 and 30. However, there were shifts in the relative abundance of these phyla from D7 to D30 microbiota (D7 vs. D30: Firmicutes, 62.14% vs. 24.59%; Bacteroidetes, 25.57% vs. 53.27%; Proteobacteria, 8.21% vs. 15.59%; Actinobacteria, 0.76% vs. 5.92%; and other phyla, 3.32% vs. 0.63%). PERMANOVA analyses based on Bray-Curtis distance dissimilarity matrix revealed that barn cleaning methods had limited impact (adonis *P* = 0.23, Fig. 1a) on the D7 microbial community structure and a modest impact on D30 microbiota (R^2^ = 0.02, adonis *P* < 0.01, Fig. 1b). However, there were distinct separations between D7 and D30 cecal microbiota irrespective of the barn cleaning method (Fig. 1c, adonis *P* < 0.001). The D30 microbiota had greater distance to the centroid compared with the D7 microbiota (distance to centroid = 0.48 and 0.56 for D7 and D30, respectively, FDR-adjusted *P* < 0.01), indicating that the D7 microbiota had greater homogeneity. In addition, the Shannon index indicated that barn cleaning methods had minimal impact on alpha diversity at D7 (*P* = 0.96) and D30 (*P* = 0.25); however, differences were observed between D7 and D30 microbial communities as reflected by increased Shannon diversity with age (*P* < 0.05) (Fig. 1d through f). In terms of differentially abundant taxa, LEfSe analysis revealed no taxa with differences in the relative abundance between FD and WW groups at D7 (FDR adjusted *P* > 0.05). However, at D30, *Ruminococcus torques, Faecalibacterium prausnitzii, Barnesiella viscericola*, and *Helicobacter pullorum* were enriched in the WW group, whereas *Megamonas funiformis* was higher in the FD group (FDR-adjusted *P* < 0.05, LDA >2, Fig. 2). In addition, successional changes on the cecal microbial composition were demonstrated by LEfSe analysis. Briefly, 11 and 8 bacterial families were associated with the D7 and D30 chicken cecal microbiota, respectively (Fig. S2).

**Fig 3:**
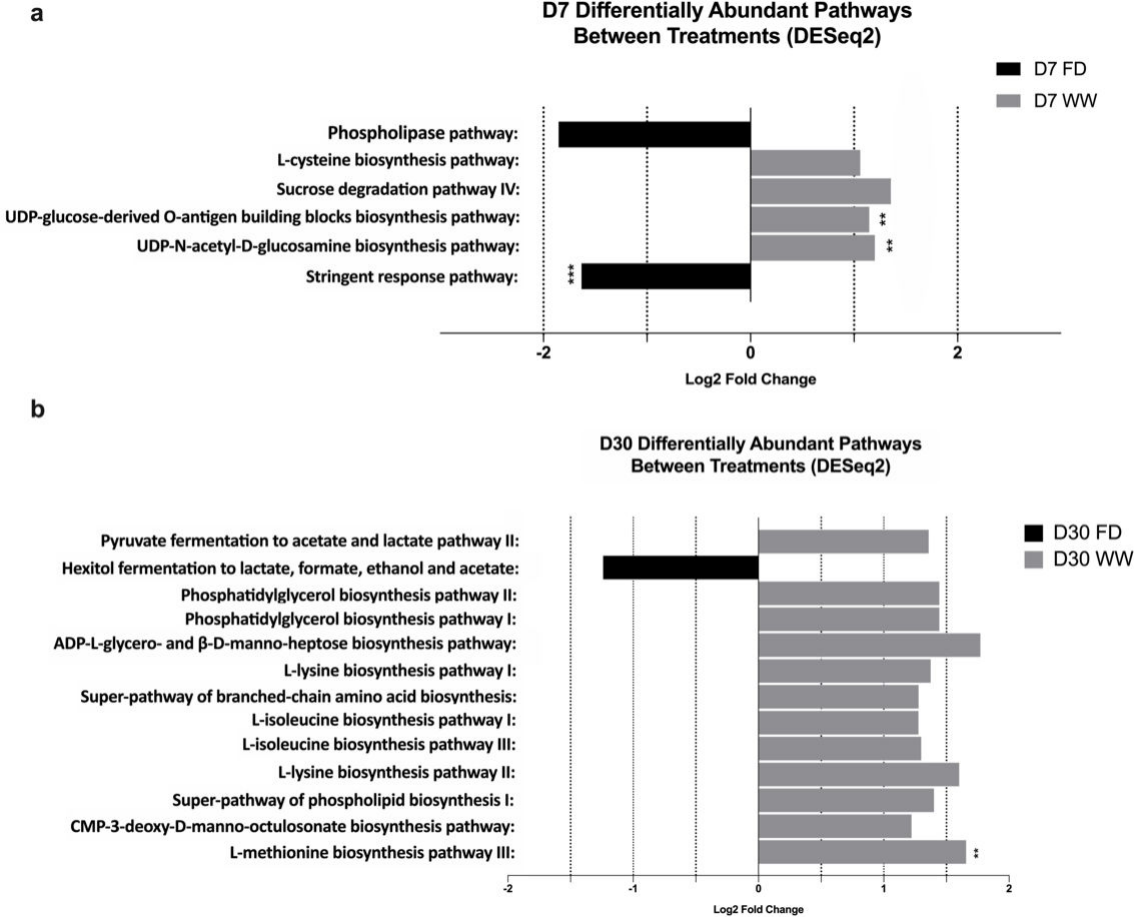
Microbial functional pathways that were significantly impacted by the barn cleaning treatments at days 7 (**a**) and 30 (**b**) revealed by DESeq2*.* The graph shows differentially abundant genetic pathways harbored by the chicken cecal microbial communities at days 7 and 30 suggested by DESeq2, respectively (FDR *P* < 0.05, log2 fold-change >1). (**a**) At day 7, the FD-derived chicken gut microbiome had enriched stringent response pathway coupled with decreased abundance of pathways linked to amino acid synthesis, saccharide degradation, and bacterial cell wall synthesis. (**b**) At day 30, the FD-derived chicken gut microbial functional capacity had decreased abundance of genetic pathways linked to multiple amino acid syntheses. D7, day 7; D30, day 30; FD, full disinfection; WW, water-wash; **, FDR *P* < 0.01; ***, FDR *P* < 0.001.

**Fig 4:**
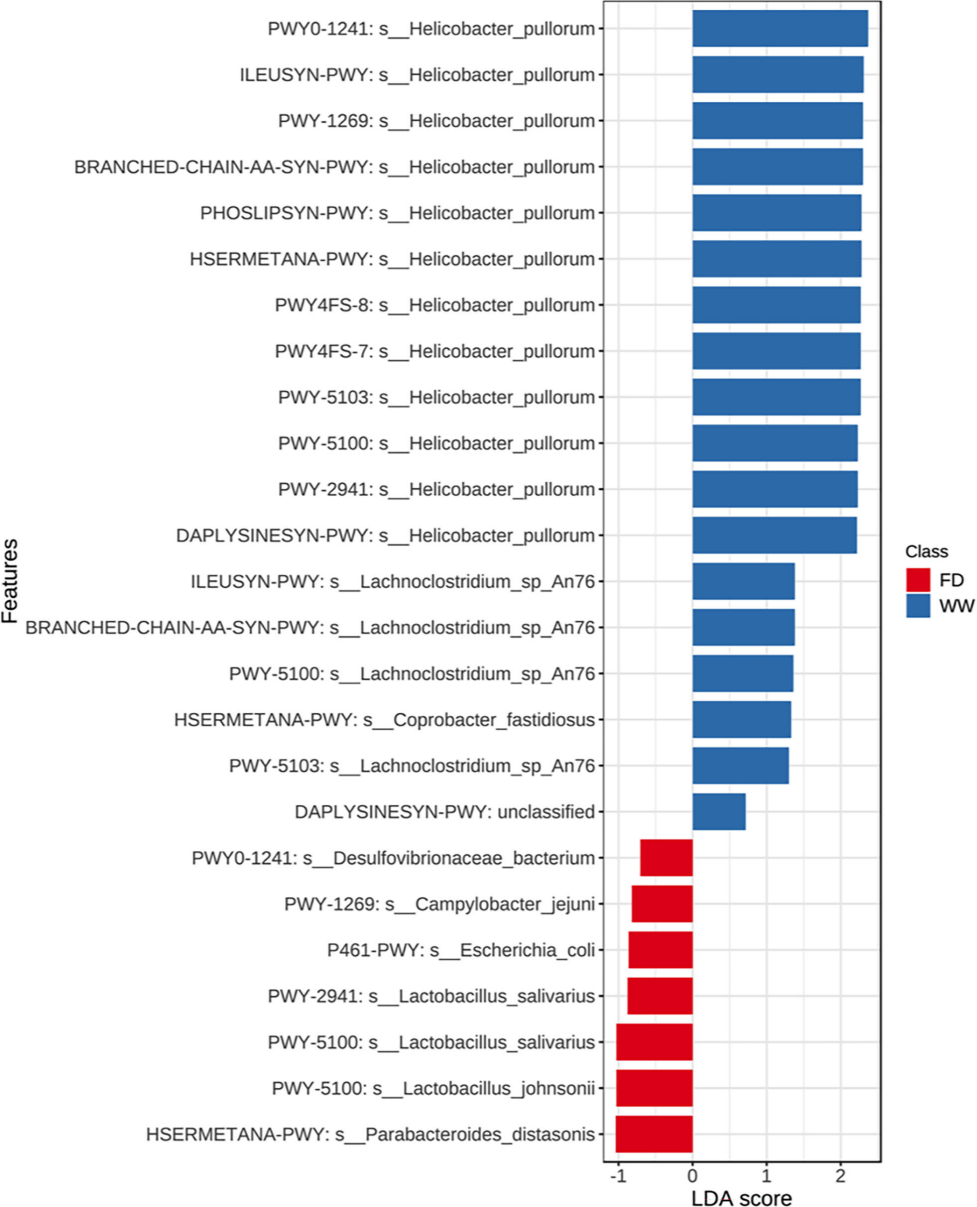
LEfSe results of metabolic pathways harbored by specific bacterial species and the association with treatments at day 30 (FDR-adjusted *P* < 0.05). LEfSe result suggested that the increased abundant pathways in the WW group were mainly contributed by *Helicobacter pullorum.* FD, full disinfection; WW, water-wash.

Cecal microbial functionalities were subsequently profiled and annotated, resulting in an average of 64.63% ± 1.18% of the total reads per sample (mean ± SEM) being mapped to the UniProt 90 database and further annotated. At D7, six pathways were altered by the barn cleaning methods (Fig. 3a, log2 fold-change > 1, FDR-adjusted *P* < 0.05), including the sucrose degradation pathway IV (PWY-5384), the L-cysteine biosynthesis pathway VI (PWY-I9), the super-pathway of UDP-glucose-derived O-antigen building blocks biosynthesis (PWY-7328), the UDP-N-acetyl-D-glucosamine biosynthesis pathway (UDPNAGSYN-PWY), the phospholipase pathway (LIPASYN-PWY), and the stringent response guanosine 3′-diphosphate 5′-diphosphate metabolism pathway (PPGPPMET-PWY). Among these altered pathways, PWY-5384 was mainly harbored by *Escherichia coli, Lactobacillus* spp., and *Bifidobacterium* spp. At D30, a series of pathways were altered by barn cleaning methods (Fig. 3b). Specifically, 12 pathways were enriched in the WW group, including the pyruvate fermentation to acetate and lactate pathway II (PWY-5100), the L-lysine biosynthesis pathway I (DAPLYSINESYN-PWY), the L-lysine biosynthesis pathway II (PWY-2941), the L-isoleucine biosynthesis pathway I (ILEUSYN-PWY), the L-methionine biosynthesis pathway III (HSERMETANA-PWY), the L-isoleucine biosynthesis pathway III (PWY-5103), the phosphatidylglycerol biosynthesis pathway I (PWY4FS-7), the phosphatidylglycerol biosynthesis pathway II (PWY4FS-8), the ADP-L-glycero- and β-D-manno-heptose biosynthesis pathway (PWY0-1241), the CMP-3-deoxy-D-manno-octulosonate biosynthesis pathway (PWY-1269), the super-pathway of phospholipid biosynthesis I (PHOSLIPSYN-PWY), and the super-pathway of branched chain amino acid biosynthesis (BRANCHED-CHAIN-AA-SYN-PWY), whereas the FD group had an enriched pathway involved in hexitol fermentation to lactate, formate, ethanol, and acetate (P461-PWY) (Fig. 3b). The total of 13 altered pathways at D30 were attributed to 62 different bacteria species. The enriched PWY-5100 in the WW cecal microbial community could be considered a consequence of an increased level of *H. pullorum* (Fig. 4, FDR-adjusted *P* < 0.05, LDA >2), whereas the contribution of *Lachnoclostridium* sp. *An76* was relatively smaller (FDR adjusted *P* < 0.05, LDA = 1.36). Indeed, the observed pathway enrichment linked to amino acid syntheses at the WW group was mainly attributed to *H. pullorum*. Compared with the modest impact on cecal microbial functional gene content by barn cleaning methods, age was shown as a strong factor affecting cecal microbial functionalities. A total of 89 pathways were significantly different between D7 and D30 cecal microbiome (Table S1, log2 fold-change > 1, FDR adjusted *P* < 0.05, DESeq2 analysis with sanitation treatments as blocking factor).

### The effect of barn cleaning practices and age on the cecal resistome

**Fig 5:**
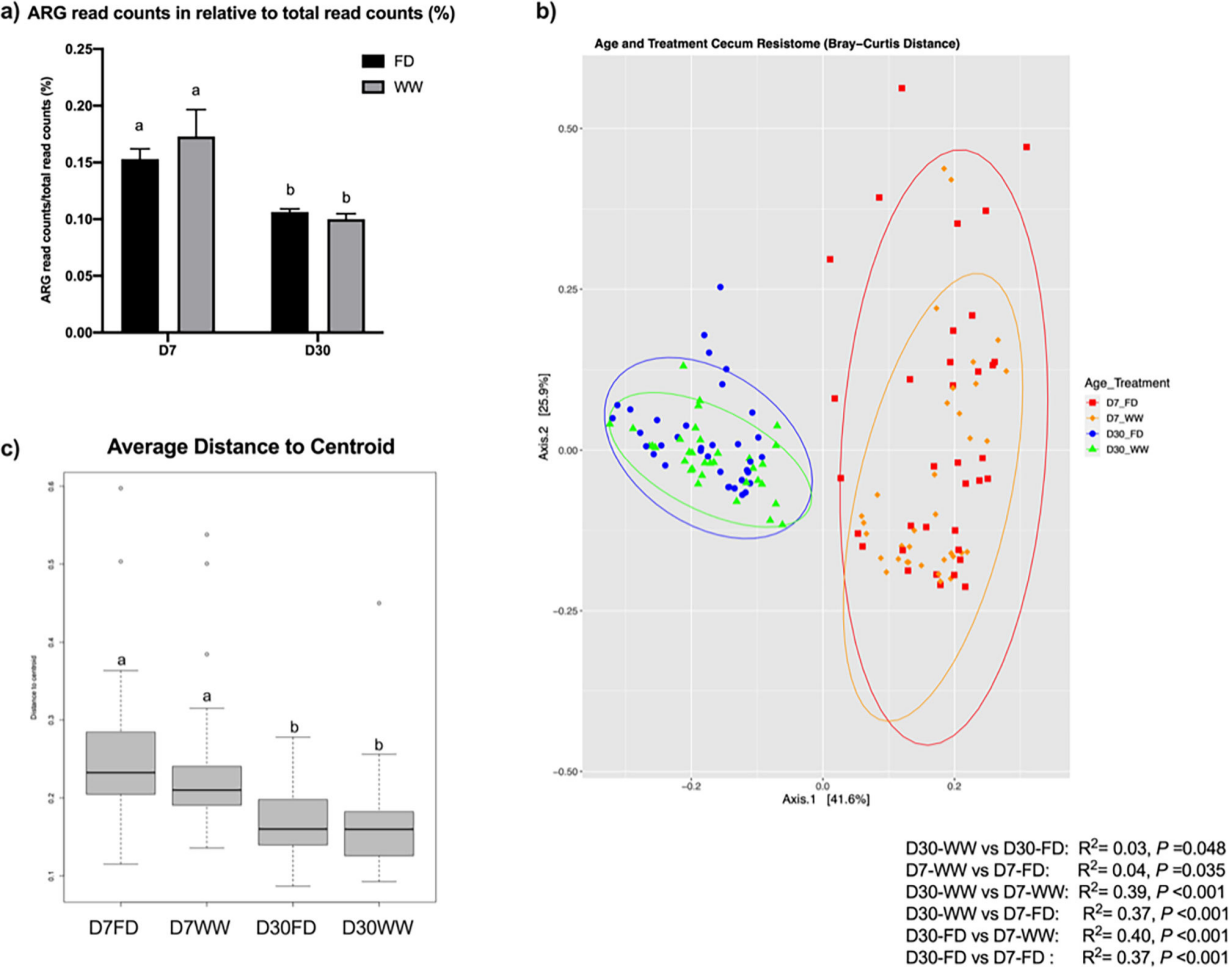
The chicken cecal resistome was affected by the barn sanitation practices and sampling time points. (**a**) ARG read counts relative to total read counts. Generally, at D7, a higher percentage of ARG read counts was detected in the chicken cecal microbiota compared to D30. (**b**) PCoA plots based on Bray-Curtis similarity distance matrix showing resistome clusters by treatments or age. Barn cleaning methods had a modest effect on the distribution of microbial resistome. Age was the main driver of the resistome pattern. (**c**) The average distance to centroid based on beta-disperse showed that the variation between resistomes was greater among the D7 chickens in comparison to the D30 chickens. ARG, antibiotic-resistant genes; D7, day 7; D30, day 30; FD, full disinfection; WW, water wash.

**Fig 6:**
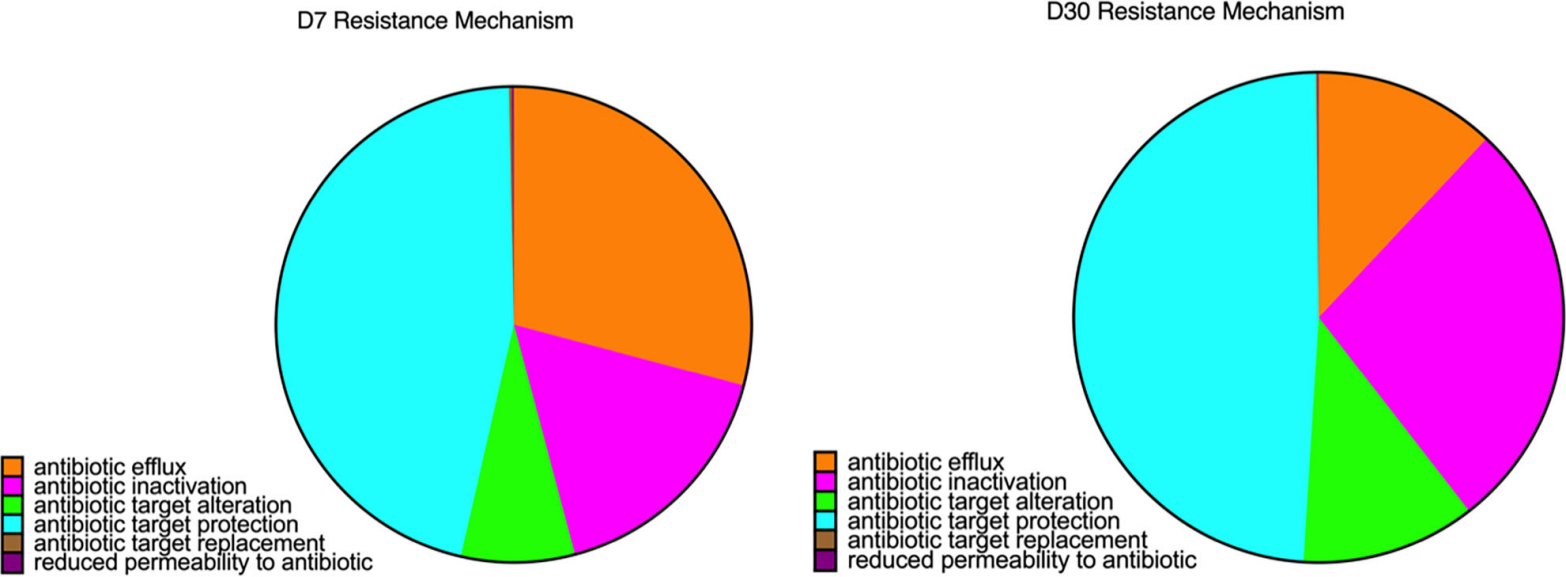
Antibiotic resistance mechanism conferred by detected ARGs in the cecal microbial resistomes of D7 and D30 chickens*.* Differences in the major antibiotic-resistant mechanisms harbored by the 7-day and 30-day chicken cecal microbial resistomes were observed. With the mechanism of antibiotic target alteration being dominant on both ages, genes conferring antibiotic efflux and antibiotic inactivation were representative of the days 7 and 30 chicken cecal resistome, respectively.

**Fig 7:**
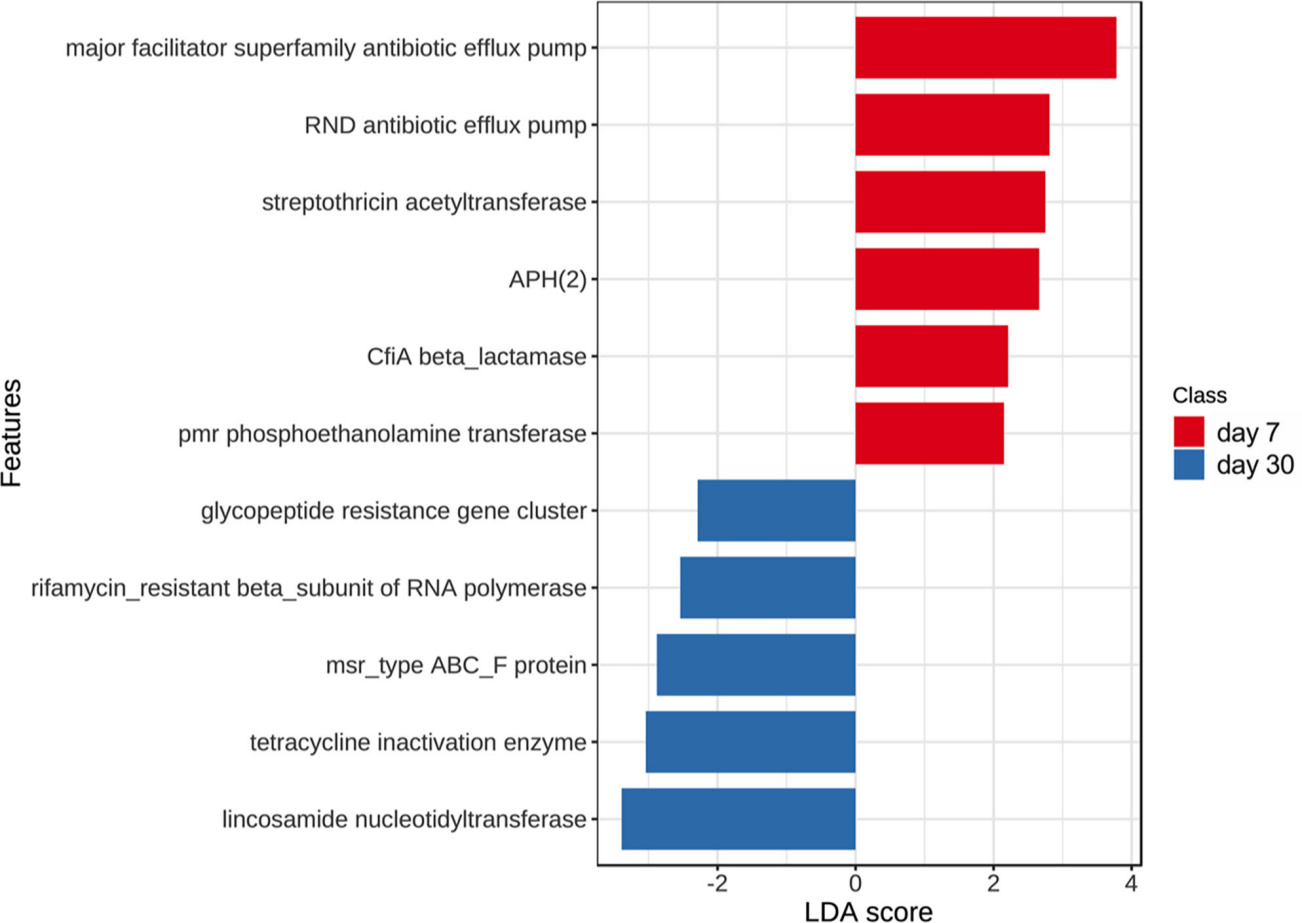
Differentially abundant cecal microbial antibiotic-resistant gene families between days 7 and 30 suggested by LEfSe*.* Graph showing antibiotic gene families associated with different ages (FDR *P* < 0.05, LDA > 2). At day 7, genes encoding antibiotic efflux pumps were representative of the chicken cecal microbial resistome. At day 30, antibiotic-resistant genes conferring antibiotic inactivation (e.g., the lincosamide nucleotidyltransferases and tetracycline inactivation enzymes) were more predominant. RND, resistance-nodulation-cell division; APH, aminoglycoside resistance gene; pmr, polymyxin resistance; msr, macrolide resistance; ABC, ATP binding cassette.

An average of 0.133% ± 0.012% of total reads per sample (mean ± SEM) were mapped to the CARD database. Both barn cleaning methods and age had an impact on the resistome (Fig. 5b, FDR *P* <0.05). Beta-dispersion analyses revealed that the cecal resistome of the 7-day-old broiler chickens had greater distance to centroid compared to that in D30 broiler chickens (*P* < 0.01), indicating more variations in cecal antimicrobial resistance at D7 (Fig. 5c). The relative abundance of total ARGs was higher at D7 compared with D30 (Fig. 5a). Overall, a total of 496 ARGs from 60 gene families and 386 ARGs from 52 gene families were identified from D7 and D30 chicken cecal microbiome, respectively. Among the detected ARGs, the tetracycline-resistant gene (*tet*) *tetW* was most abundant, followed by lincosamide nucleotidyltransferase genes (*lnu*) *lnuC*, *tet(34)*, *tet(W/N/W)*, *tetQ*, the erythromycin ribosomal methylation 23S ribosomal RNA methyltransferase (*erm*) *ermB*, aminoglycoside resistance gene (*APH*) *APH(3)-IIIa*, *tetO*, and *tet32.* In terms of gene families, tetracycline-resistant ribosomal protection proteins (RPPs) family was most abundant, followed by the *lnu* family and the major facilitator superfamily (MFS) antibiotic efflux pump (multi-drug-resistant) family. These ARG families collectively accounted for over 90% of the broiler chicken cecal resistome (92.08% ± 11.03% mean ± SEM). At D7, the most abundant ARG families in the cecal resistomes were the tetracycline-resistant RPP family, the MFS antibiotic efflux pump family, the resistance-nodulation-cell division (RND) antibiotic efflux pump (multi-drug resistant), the *erm* family, and the *lnu* family. Although at D30, the top five abundant gene families were the tetracycline-resistant RPP followed by the *lnu* family, the MFS antibiotic efflux pump family, the aminoglycoside nucleotidyltransferase (*ant*) family *ant(6)* (aminoglycoside-resistant), and the *erm* family. When characterizing the resistance mechanism and distribution of the ARGs, we discovered that antibiotic target protection, which mainly consisted of tetracycline resistance via tetracycline-resistant RPPs, was the most common mechanism of antibiotic resistance. In addition, antibiotic efflux and antibiotic inactivation were the second most common resistance mechanism found at D7 and D30, respectively (Fig. 6). LEfSe results supported the successional change of the chicken cecal resistome (Fig. 7, FDR *P* < 0.05, LDA > 2). In accordance with the different antibiotic mechanisms between D7 and D30, LEfSe analysis identified that 11 ARG gene families were associated with the age of the broiler chickens (Fig. 7). Particularly, the MFS antibiotic efflux pumps and the RND antibiotic efflux pump showed the highest LDA score at D7, indicating that antibiotic efflux plays a key role in the early life cecal resistome. Although on D30, AMR gene families conferring antibiotic inactivation, such as the *lnu* family and the tetracycline inactivation enzyme, had increased predominance.

**Fig 8:**
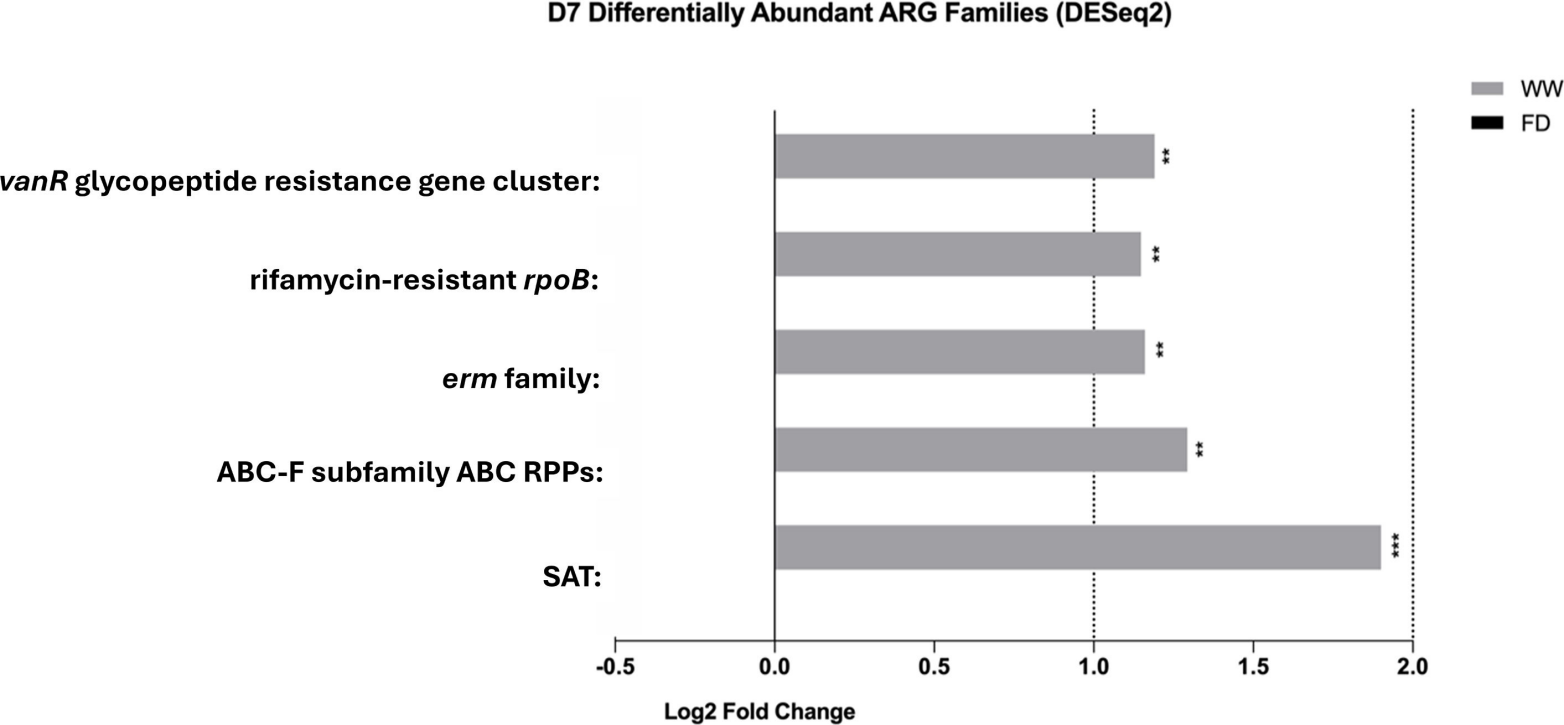
Differentially abundant antibiotic-resistant gene families between FD and WW at D7 suggested by DESeq2*.* The graph shows differentially abundant antibiotic-resistant gene families between barn sanitation practices at day 7 suggested by DESeq2 (FDR *P* < 0.05, Log2 fold change > 1). Some persistent ARG gene families (e.g., *erm* family) were enriched in the WW-derived chicken cecal microbiome at day 7. ARG, antibiotic-resistant gene; *vanR*, vancomycin-resistant gene R component; *rpoB,* gene encoding β-subunit of bacterial RNA polymerase; *erm,* 23S ribosomal RNA methyltransferase; ABC-F subfamily ABC RPPs, ABC-F ATP-binding cassette ribosomal protection protein genes; SAT, Streptogramin A acetyltransferase genes, FD, Full disinfection; and WW, Water-wash; ***, *P* < 0.001; **, *P* < 0.01.

**Fig 9:**
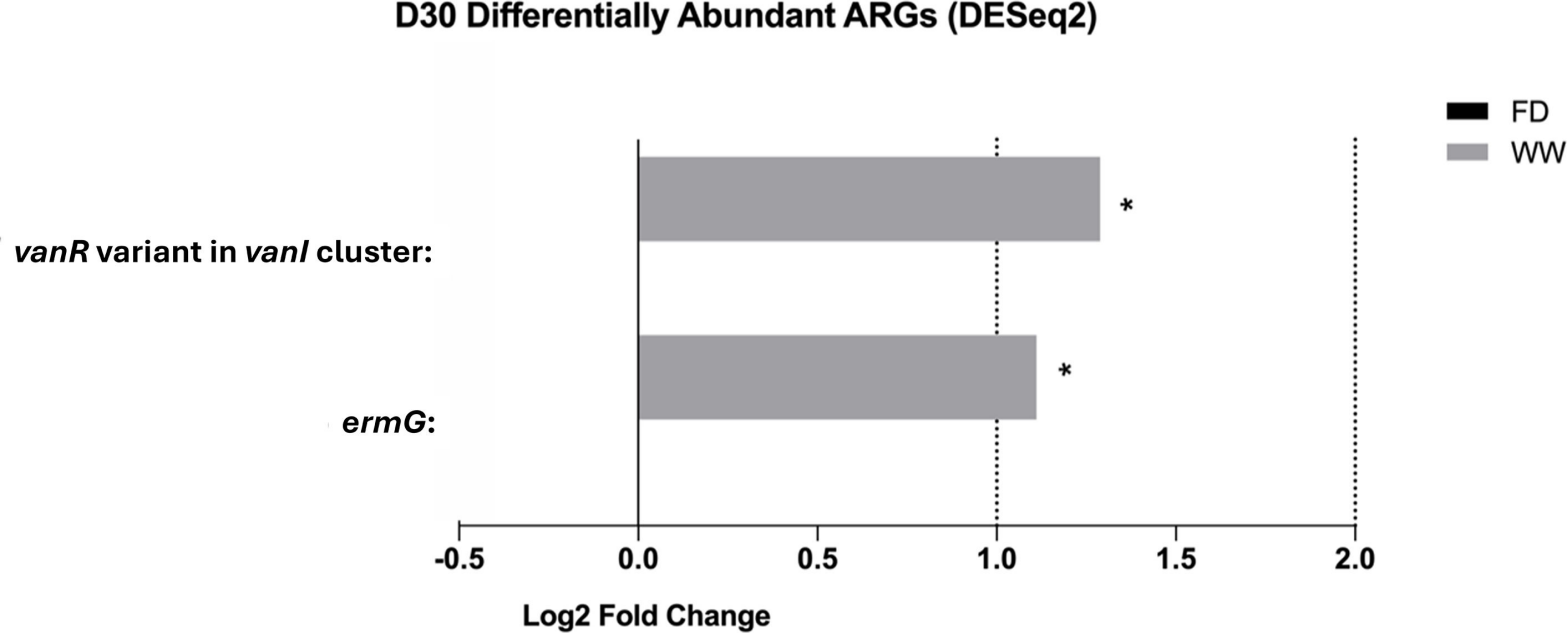
Differentially abundant antibiotic-resistant gene between FD and WW at D30 suggested by DESeq2*.* The graph shows differentially abundant antibiotic-resistant genes between barn sanitation practices at day 30 suggested by DESeq2 (FDR *P* < 0.05, Log2 fold change > 1). Compared to the impact of barn cleaning practices at day 7, the effects on the 30-day chicken gut microbial resistome were relatively smaller. On the gene level, *ermG* and *vanR* variants in the *vanI* cluster were enriched by the WW treatment. ARG, antibiotic-resistant gene; *vanR*, vancomycin-resistant gene; *erm,* 23S ribosomal RNA methyltransferase genes; FD, Full disinfection; and WW, Water-wash; *, *P* < 0.05.

Compared with the age effect, barn cleaning methods had a modest impact on the chicken resistome profile. Specifically, five AMR gene families including the *erm* family, rifamycin-resistant beta-subunit of RNA polymerase (*rpoB*), streptogramin vat acetyltransferase, ATP-binding cassette (ABC)-F subfamily RPPs (macrolide- and lincosamide-resistant), and *vanR* glycopeptide resistance gene cluster were found to be more abundant in the D7-WW treatment compared with the D7-FD treatment (Fig. 8, FDR *P* < 0.05). At the gene level, *cprR* (peptide antibiotic-resistant)*, ermB* (macrolide-, lincosamide-, and streptogramin-resistant, MLS-resistant)*, lnuA* and *lnuB* (lincosamide-resistant)*, lsaE* (pleuromutilin-, streptogramin-, and lincosamide-resistant)*, oleB* (macrolide-resistant)*, tet(L)* and *tetM* (tetracycline-resistant)*, vatE* (streptogramin-resistant)*, Vibrio anguillarum* chloramphenicol acetyltransferase gene (phenicol-resistant), the *Bifidobacterium bifidum ileS* (mupirocin-resistant), the *Bifidobacterium adolescentis rpoB* (rifampicin-resistant), and *vanR* variants in the *vanA*, *vanG*, and *vanL* clusters (vancomycin-resistant) were more abundant in the D7-WW group. At D30, the impacts of cleaning methods on the chicken gut microbiome were subtle, indicating that the barn cleaning practices had greater impacts in younger chickens. No differentially abundant ARG gene families were identified between FD and WW by DESeq2. However, genes including *ermG* (MLS-resistant) and *vanR* variant in *vanI* cluster (vancomycin-resistant) were enriched in the D30-WW group (Fig. 9).

## DISCUSSION

To date, limited information is available regarding how barn cleaning methods affect the chicken cecal microbiota. Using 16S rRNA sequencing technique, we previously reported that at D30, the genus *Helicobacter* was enriched in the WW group (24). Due to initial resource limitations, in-depth shotgun metagenomic sequencing could not be performed alongside our earlier phenotypic and amplicon-based sequencing. This study, therefore, builds on those findings, enabled by additional funding obtained after our prior publication (24). In the current study, functional genetics analyses suggested that the barn cleaning methods altered the functional gene content of the chicken cecal microbiota. The exposure to FD at 1 day of life may impact the assembly of the early cecal microbiota of chicks and thereby lead to altered microbial functional gene content observed later in life, which possesses decreased genetic potential for amino acid and SCFA synthesis. In addition, we confirmed that chicken cecal *H. pullorum* harboring genes linked to SCFA and amino acid production was higher in the WW treatment. In the present study, the sample size of each production flock was set to five broiler chickens at each age. While increasing the sample size within each flock may strengthen the conclusion, it is also important to recognize that previous microbiome studies observed cohousing effects, which can lead to misinterpretation of data (34). In the current study, we also observed a flock effect of the microbiome data (data not shown). In this case, increasing the number of observations of barns could help eliminate bias introduced by flock effect. Furthermore, our experimental design included both FD and WW treatments for each barn, which helps control for biases related to cohousing and management practices. Shotgun metagenomic sequencing of the samples provided taxonomic information to the species level with confidence. In the current study, the WW group exhibited an increased relative abundance of *H. pullorum, F. prausnitzii, B. viscericola,* and *R. torques.* Conversely, there was a notable decrease in the relative abundance of *M. funiformis* in the WW group compared to the FD group*.* While *H. pullorum* has been suggested to be an opportunistic pathogen (35), its precise role in poultry and its pathogenicity to human and chickens remains unclear. Importantly, *H. pullorum* is increasingly recognized as an emerging zoonotic organism that can colonize poultry and may be transmitted to humans through contaminated meat when undercooked, where it has been associated with human colitis and may exacerbate hepatitis C virus infection (36, 37). However, direct causal links between *H. pullorum* and other pathogens and diseases in poultry or humans remain limited and not fully elucidated. Given its enrichment in the WW group, this finding raises a potential food safety concern that warrants caution. Future studies are needed to investigate the prevalence of viable *H. pullorum* in poultry products, its persistence through processing, and the actual risk it may pose to human health. Similar to the current study, a previous study showed that the prevalence of *Faecalibacterium* increased in chicken ceca from the group treated by recycled litter compared with the fresh litter group, which was concluded as a beneficial effect of recycled litter (38). As a commensal member also found to be enriched in conventionally raised chickens (39), *F. prausnitzii* was identified as a promoter of epithelial health for its ability to produce metabolites such as butyrate (40). Moreover, it was found to show anti-inflammatory activity in broilers (41). *B. viscericola* has also been characterized as an important commensal in barn-raised chickens (42–44). In addition, members from *Ruminococcus* have been characterized as important butyrate producers in the gut that degrades mucins (40, 45); however, there is limited information available concerning the role of *R. torques* in the chicken gut.

The current study unveiled alterations in a range of microbial metabolic pathways within the chicken ceca as a result of barn cleaning practices. For example, at D7, the stringent response pathway (PPGPPMET-PWY) was enriched in the cecal microbiome of the FD group, which was primarily harbored by *E. coli.* The stringent response regulates genes involved in response to nutrient starvation or environmental stresses (46). It is important for bacterial virulence and persistence in the environment (e.g., resistance to antimicrobials) for a variety of taxa (46) and is used by *Bacteroides* to shift from growth to stasis (47). Furthermore, a previous study revealed that the stringent response can induce microbes to the viable but nonculturable state, a state which has strong tolerance to environmental stresses with minimum nutrient requirement (48). In this sense, it is reasonable to speculate the enrichment of the stringent pathway might indicate that the chemical disinfection selects for bacteria harboring PPGPPMET-PWY to tolerance harsh environmental conditions (49).

Furthermore, it is interesting to note that the early enrichment of PPGPPMET-PWY in the FD-derived gut microbiome at D7 coincided with a decreased capacity in amino acid synthesis, including branched-chain amino acid synthesis, observed at D30. Although causality cannot be established from the present data, this temporal pattern raises the hypothesis that early life stress responses might have long-term effects on microbiome development. For example, ppGpp (guanosine pentaphosphate and guanosine tetraphosphate) is important in modulating the activity of CodY, a DNA binding-protein that serves as an important transcription regulator in some low-GC, gram-positive bacteria (50). During stress, the accumulation of ppGpp lowers the intracellular GTP pool, leading to the inactivation of CodY (49). Meanwhile, it is also known that CodY regulates biosynthesis of branched-chain amino acids (51), leucine and isoleucine (52). We speculate that in this study, early exposure to chemical disinfectants could have triggered such stringent response-related regulatory shifts, potentially contributing to the observed later-life reduction in branched-chain amino acid biosynthesis potential. Therefore, future study focusing on transcriptional regulations will be needed to test this proposed linkage and to further elucidate the interplay between chemical disinfection, bacterial stringent responses, amino acid synthesis, and microbiome development.

At day 30, microbial metabolic pathways linked to SCFA production (acetate and lactate) and amino acid biosynthesis (L-methionine, L-lysine, L-isoleucine, and branched-chain amino acids) were enriched in the WW group. This suggests that the WW treatment may have led to an enhancement in the nutrient utilization functional gene content of the gut microbial community at day 30. In the current study, PWY-5100 was mainly harbored by *H. pullorum* in both the FD and WW group*,* and to a lesser extent, *Lachnoclostridium* sp. *An76* and *Lactobacillus salivarius*. While the specific impact of the enrichment of the pathway in the WW group remains to be confirmed by further study, previous research has highlighted its potential relevance in chicken gut health, particularly in host pathogen defense. Previously, Gong et al. reported that chickens infected by *Clostridium perfringens* had a significant inhibition of the pyruvate fermentation to acetate and lactate pathway II (PWY-5100) in the cecal microbiome, which was restored by the supplementation of probiotic *L. plantarum* (53). It is important to note that our study did not include a bacterial challenge. Therefore, while the enrichment of PWY-5100 in the WW-derived microbiome is interesting, further studies on assessing how the increased capacity of PWY-5100 affects digesta SCFA or chicken performance with pathogen challenges would be needed.

Interestingly, PWY-1269 was shown to be mainly harbored by *H. pullorum* and *C. jejuni* in the chicken gut microbiome of the WW and FD, respectively. PWY-1269 encodes genes that produce acid sugar 3-deoxy-α-D-manno-2-octulosonate, which is a component of bacterial lipopolysaccharides (LPS) (54–56). We have previously reported that *C. jejuni* was decreased in the D30-WW group (24), which was consistent with the result from functional analyses in the current study as reflected by decreased *C. jejuni-*derived LPS genes in WW-cecal microbiome. Regarding the ARG profiles in the chicken cecal microbiota, in the current study, the most abundant ARGs detected confer resistance to tetracycline [*tetW, tet(42), tet(W/N/W), tetQ, tetO,* and *tet(32)*], MLS (*lnuC, ermB*), and aminoglycoside (*APH(3)-IIIa*), whereas β-lactam resistance was found in relatively low prevalence. Tetracycline-resistance genes, particularly *tetW,* were frequently detected in environments related to livestock farming (57–61), as well as bacteria isolated from the chicken gut (62). Previously, Munk et al. reported that the majority of ARGs in chicken fecal samples collected from multiple European countries were tetracycline-, aminoglycoside-, and MLS-resistant genes (18). Similarly, chicken fecal samples collected from China were high in aminoglycoside, tetracycline, MLS, and β-lactam resistance (63). The low abundance of β-lactam resistant genes observed in this study may link to the prohibition of prophylactic use of β-lactam in poultry farming since 2018 (64), whereas β-lactam was reported to be one of the most commonly used antibiotics in poultry production in China (65).

Our observations indicate that both barn cleaning methods and the age of the chickens influenced the microbial resistome. Age emerged as the primary factor driving alterations in the resistome. In line with previous studies (66, 67), we observed that the barn-cleaning effects on the gut resistome decreased with increasing age (66, 67).

While our initial expectation was that disinfection might enhance the selection of antibiotic resistance genes (ARG), our results indicate that disinfection is linked to a reduced abundance of ARG. This finding suggests that the impact of disinfection on ARG transmission between flocks merits further evaluation as a potential strategy for controlling ARG spread. It has been reported that poultry litter harbored high densities of AMR bacteria and antibiotic residuals (68–70). Although poultry litter was removed in the current study, without chemical disinfectants, WW might preserve bacteria carrying ARGs. Therefore, it is reasonable to assume that compared to WW, the chemical disinfectants used in the current study may be more effective in controlling ARGs. Notably, at both D7 and D30, ARGs from the *erm* family and the glycopeptide resistant gene cluster (*vanR*) were depleted by the disinfection. Currently, more than 30 *erm* genes have been characterized, and a number of them (e.g., *ermB, ermC, ermG, ermF,* and *ermX*) were frequently detected in livestock farming-related environments (71–73). Frequent horizontal transfer of *erm* genes through mobile genetic elements has been reported within the gut microbiota (74, 75), as well as between intestinal and environmental bacteria (76, 77), accounting for their distribution among diverse taxa. Additionally, *erm* genes were found to persist stably both in the gut and in the environment 2–3 years after the removal of antibiotic selection pressure (73, 78). Furthermore, *erm* genes were also known to spread through poultry dust (61, 79), indicating possibilities of persistent *erm* residues from the previous production cycles. The *vanS/vanR*, two-component regulatory system, is important in activating and regulating transcription of the glycopeptide gene cluster. Vancomycin-resistant genes have been detected in poultry farms (80) and products (81). Similar to *erm* genes, a recent study revealed a glycopeptide-resistant gene cluster persisting in the environment for 20 years (82). In addition, glycopeptide resistance gene clusters are also highly transferable via plasmids (81, 83), making it difficult to identify the main carriers.

In the present study, we found that the relative abundance of total ARGs was higher in the ceca of D7 chickens compared to D30. Previously, Lebeaux et al. reported similar results in human infants showing that the overall relative abundance of ARGs was higher at 6 weeks than 1 year (84). We noted a heightened proportion of genes encoding antibiotic target alteration coupled with a diminished proportion of genes encoding antibiotic efflux in the chicken gut resistome from day 7 to day 30. The successional change of the chicken gut microbiota may have contributed to the alteration of cecal ARG composition. LEfSe analysis suggested that the family *Lachnospiraceae* and *Enterobacteriaceae* were strong biomarkers of the chicken cecal microbiota at D7. In addition, Spearman correlation revealed that the relative abundance of the MFS antibiotic efflux pump family was positively correlated to *Lachnospiraceae* (*Lachnoclostridium* sp *An76*), further supporting the role *Lachnospiraceae* may play in the gut microbial resistome (Fig. S3). Juricova et al. compared ARG sequences and bacterial genomes and reported that the family *Lachnospiraceae* was an important reservoir for MFS antibiotic efflux pumps (62). Thus, the predominance of the MFS antibiotic efflux pumps detected in the D7-cecal resistome may be partially explained by the predominance of *Lachnospiraceae* in early life. In addition, *Enterobacteriaceae* (*E. coli* and *E. albertii*) were shown to be positively correlated with the abundance of the RND antibiotic efflux pumps (Fig. S3). *Enterobacteriaceae* are known to harbor the RND antibiotic efflux pumps (85, 86), and many RND genes were enriched in *Enterobacteriaceae* in chicken litter and cloacal samples (67). Thus, the enriched RND antibiotic efflux pump family at D7 may be a consequence of the early colonization of *Enterobacteriaceae*. In addition, among all taxa, *E. coli* was associated with the highest number of ARG families (Fig. S3). Interestingly, in the human infant resistome study, Lebeaux et al. concluded that the human early-life resistome composition was primarily driven by *E. coli* (84).

The D30 resistome was associated with antibiotic inactivation genes, particularly genes encoding LNUs (mainly *lnuC* gene) and tetracycline inactivation enzymes (mainly *tetX* gene and its variants). Lincosamide-resistant gene *lnuC* has been identified in the genus *Streptococcus* (87) and was shared extensively between different phyla (88). Emerging evidence also showed that *C. jejuni and C. coli* harbor *lnuC* (89–92). The increased relative abundance of *Streptococcaceae* and *Campylobacteraceae* at D30 may partially explain the enriched *lnu* family observed in older chickens. Consistent with the effect of age, *lnu* genes were also more predominant in adult cattle compared to calves (66). Interestingly, *Bacillus subtilis* was positively correlated to the *lnu* family (Fig. S3). *B. subtilis* has exhibited an ability to naturally activate the competence state and uptake foreign DNA (93), making it a potential carrier of *lnu* genes. However, the degree to which microbial compositions affect ARG composition is still unclear (94), especially in the case of highly mobile ARGs such as *lnuC*.

The present study is subject to several limitations that should be considered when interpreting the findings. First, although a cross-over design across seven broiler barns was used for multiple production cycles, only five birds per barn per time point were sampled. While this approach allowed us to capture data from multiple barns and time points, the relatively small number of biological replicates per flock provides only a glimpse of the nature of the poultry gut microbiome within this broader environment. In particular, it may have led to underestimation of subtle differences in the functional and ARG profiles. Sampling depth in the current study was determined by constraints such as sequencing costs and challenges of field-scale sampling. We acknowledge that studies with higher per-group sample sizes would be better positioned to detect smaller effect sizes and to provide more comprehensive representation of the microbiomes. Second, due to insufficient environmental DNA extracted from environmental samples, the microbiome of the initial environmental samples, such as litter and surface swabs, was not assessed. Given poultry litter and barn surfaces can retain substantial ARG reservoirs after cleaning and disinfection, it is possible that residual ARGs carried by bacteria in the barn environment acted as sources for re-colonization or re-seeding of resistomes in subsequent flocks. The absence of these environmental data limits our ability to disentangle direct impacts of disinfection from environmental re-seeding and constrains interpretation of the long-term efficacy of the intervention. Third, the resistome analyses relied on shotgun metagenomic sequencing and *in silico* analyses. While this culture-independent approach provides valuable information, it is difficult to confirm whether these genes are transcriptionally active, translated into functional proteins, or confer phenotypic resistance within the gut microbial community. Likewise, metabolic pathway prediction and analyses derived from metagenomics data do not distinguish between active and inactive metabolic responses. Last but not least, while our findings suggest that FD may modestly reduce certain ARG carriage in the chicken gut, the present data does not directly demonstrate whether this translates into reduced ARG dissemination through the production chain, for example, in the meat processing plants or in final meat products.

Therefore, future work should aim to increase the number of birds sampled per barn to improve statistical power and sensitivity for the detection of less prevalent microbial or resistance determinants. In addition, increased volume of environmental sampling to obtain enough material for comprehensive environmental ARG surveillance is warranted to capture the full dynamics of resistance gene persistence and reintroduction. Furthermore, culture-based studies assessing antibiotic/disinfectant susceptibility and ARG mobility targeting isolates of keystone taxa of commercially raised broiler chickens are of great importance. Complementary functional validation using approaches such as metatranscriptomics or reverse transcription-qPCR targeting a selection of resistance genes, proteomic or metabolomics profiling will also be essential for linking genomic potential to gene expression, protein activity, and metabolic function.

### Conclusion

This is the first study to report that the impact of disinfectants in broiler production on microbial functional gene content and resistome. We showed that barn chemical disinfection may alter the composition of the chicken gut microbiota and thereby lead to decreased microbial functional gene content for amino acid and SCFA metabolism. Conversely, although differences were modest, FD may be beneficial through lowering abundance and diversity of ARGs. For broiler chicken production, the current study presents a practical trade-off. While chemical disinfection may help slightly mitigate ARG carriage, especially those that are persistent, it also reduces the gut microbial functional gene content, which may have longer-term implications for flock performance. Considering current evidence, prioritizing a robust gut microbiome may outweigh the subtle benefit of ARG suppression, particularly in the current production settings where prophylactic antibiotic usage is already strictly controlled. Therefore, frequent FD treatment between every flock may not be universally justified if the main goal is to minimize ARG burden, as WW methods also support beneficial microbiome functions and do not compromise flock performance (24). Future on-farm decisions should consider both antimicrobial stewardship and the importance of a robust gut microbiome. Additional studies incorporating longer-term production outcomes and flock health monitoring are warranted to refine recommendations.

## Data Availability

The sequences from the current study have been submitted to the NCBI Sequences Read Archive under the BioProject ID: PRJNA1108021.

## SUPPLEMENTAL MATERIAL

Supplemental material spectrum.03737-25-s0001.docx Fig. S1 to S3; Table S1.

ASM does not own the copyrights to Supplemental Material that may be linked to, or accessed through, an article. The authors have granted ASM a non-exclusive, world-wide license to publish the Supplemental Material files. Please contact the corresponding author directly for reuse.

## References

[1] Van Boeckel TP, Glennon EE, Chen D, Gilbert M, Robinson TP, Grenfell BT, Levin SA, Bonhoeffer S, Laxminarayan R. 2017. Reducing antimicrobial use in food animals. Science 357:1350–1352. doi:10.1126/science.aao1495 28963240 PMC6510296

[2] Qian X, Gu J, Sun W, Wang X-J, Su J-Q, Stedfeld R. 2018. Diversity, abundance, and persistence of antibiotic resistance genes in various types of animal manure following industrial composting. J Hazard Mater 344:716–722. doi:10.1016/j.jhazmat.2017.11.020 29154097

[3] Murray CJL, Ikuta KS, Sharara F, Swetschinski L, Robles Aguilar G, Gray A, Han C, Bisignano C, Rao P, Wool E, et al.. 2022. Global burden of bacterial antimicrobial resistance in 2019: a systematic analysis. The Lancet 399:629–655. doi:10.1016/S0140-6736(21)02724-0 PMC8841637 35065702

[4] Kampf G. 2018. Biocidal agents used for disinfection can enhance antibiotic resistance in gram-negative species. Antibiotics (Basel) 7:110. doi:10.3390/antibiotics7040110 30558235 PMC6316403

[5] McNamara PJ, Levy SB. 2016. Triclosan: an instructive tale. Antimicrob Agents Chemother 60:7015–7016. doi:10.1128/AAC.02105-16 27736758 PMC5118979

[6] Zeng J, Li Y, Jin G, Su J-Q, Yao H. 2022. Short-term Benzalkonium Chloride (C _12_) exposure induced the occurrence of wide-spectrum antibiotic resistance in agricultural soils. Environ Sci Technol 56:15054–15063. doi:10.1021/acs.est.2c04730 36069710

[7] Zhang Y, Gu AZ, He M, Li D, Chen J. 2017. Subinhibitory concentrations of disinfectants promote the horizontal transfer of multidrug resistance genes within and across genera. Environ Sci Technol 51:570–580. doi:10.1021/acs.est.6b03132 27997135

[8] Kim M, Weigand MR, Oh S, Hatt JK, Krishnan R, Tezel U, Pavlostathis SG, Konstantinidis KT. 2018. Widely used Benzalkonium chloride disinfectants can promote antibiotic resistance. Appl Environ Microbiol 84:e01201-18. doi:10.1128/AEM.01201-18 29959242 PMC6102991

[9] Gadea R, Fernández Fuentes MÁ, Pérez Pulido R, Gálvez A, Ortega E. 2017. Effects of exposure to quaternary-ammonium-based biocides on antimicrobial susceptibility and tolerance to physical stresses in bacteria from organic foods. Food Microbiol 63:58–71. doi:10.1016/j.fm.2016.10.037 28040182

[10] Slifierz MJ, Friendship RM, Weese JS. 2015. Methicillin-resistant Staphylococcus aureus in commercial swine herds is associated with disinfectant and zinc usage. Appl Environ Microbiol 81:2690–2695. doi:10.1128/AEM.00036-15 25662976 PMC4375337

[11] Hall MC, Duerschner J, Gilley JE, Schmidt AM, Bartelt-Hunt SL, Snow DD, Eskridge KM, Li X. 2021. Antibiotic resistance genes in swine manure slurry as affected by pit additives and facility disinfectants. Sci Total Environ 761:143287. doi:10.1016/j.scitotenv.2020.143287 33168251

[12] Yoon Y, Chung HJ, Wen Di DY, Dodd MC, Hur H-G, Lee Y. 2017. Inactivation efficiency of plasmid-encoded antibiotic resistance genes during water treatment with chlorine, UV, and UV/H2O2. Water Res 123:783–793. doi:10.1016/j.watres.2017.06.056 28750328

[13] Jurburg SD, Brouwer MSM, Ceccarelli D, van der Goot J, Jansman AJM, Bossers A. 2019. Patterns of community assembly in the developing chicken microbiome reveal rapid primary succession. Microbiologyopen 8:e00821. doi:10.1002/mbo3.821 30828985 PMC6741130

[14] Ocejo M, Oporto B, Hurtado A. 2019. 16S rRNA amplicon sequencing characterization of caecal microbiome composition of broilers and free-range slow-growing chickens throughout their productive lifespan. Sci Rep 9:2506. doi:10.1038/s41598-019-39323-x 30792439 PMC6385345

[15] Feng Y, Zhang M, Liu Y, Yang X, Wei F, Jin X, Liu D, Guo Y, Hu Y. 2023. Quantitative microbiome profiling reveals the developmental trajectory of the chicken gut microbiota and its connection to host metabolism. iMeta 2:iMeta doi:10.1002/imt2.105 PMC10989779 38868437

[16] Course CE, Boerlin P, Slavic D, Vaillancourt J-P, Guerin MT. 2021. Factors associated with Salmonella enterica and Escherichia coli during downtime in commercial broiler chicken barns in Ontario. Poult Sci 100:101065. doi:10.1016/j.psj.2021.101065 33765489 PMC8008170

[17] Yang J, Tong C, Xiao D, Xie L, Zhao R, Huo Z, Tang Z, Hao J, Zeng Z, Xiong W. 2022. Metagenomic insights into chicken gut antibiotic resistomes and microbiomes. Microbiol Spectr 10:e01907-21. doi:10.1128/spectrum.01907-21 35230155 PMC9045286

[18] Munk P, Knudsen BE, Lukjancenko O, Duarte ASR, Van Gompel L, Luiken REC, Smit LAM, Schmitt H, Garcia AD, Hansen RB, et al.. 2018. Abundance and diversity of the faecal resistome in slaughter pigs and broilers in nine European countries. Nat Microbiol 3:898–908. doi:10.1038/s41564-018-0192-9 30038308

[19] Wu X, Zhang Z, Xiang R. 2025. Whole genome analysis reveals the distribution and diversity of plasmid reservoirs of NDM and MCR in commercial chicken farms in China. Microbiol Spectr 13:e02900-24. doi:10.1128/spectrum.02900-24 40488461 PMC12210879

[20] Paul SS, Rama Rao SV, Hegde N, Williams NJ, Chatterjee RN, Raju MVLN, Reddy GN, Kumar V, Phani Kumar PS, Mallick S, Gargi M. 2022. Effects of dietary antimicrobial growth promoters on performance parameters and abundance and diversity of broiler chicken gut microbiome and selection of antibiotic resistance genes. Front Microbiol 13:905050. doi:10.3389/fmicb.2022.905050 35783415 PMC9244563

[21] Huang Y, Zong S, Xu D, He J, Zhang Y, Qian M, Li Y, Guo B, Han J, Qu D. 2025. Metagenomic analysis reveals differences in antibiotic resistance and transmission risks across various poultry farming models. Science of The Total Environment 980:179519. doi:10.1016/j.scitotenv.2025.179519 40300492

[22] Pan Y, Zeng J, Zhang L, Hu J, Hao H, Zeng Z, Li Y. 2024. The fate of antibiotics and antibiotic resistance genes in large-scale chicken farm environments: preliminary view of the performance of national veterinary antimicrobial use reduction action in Guangdong, China. Environ Int 191:108974. doi:10.1016/j.envint.2024.108974 39186902

[23] Temmerman R, Ghanbari M, Antonissen G, Schatzmayr G, Duchateau L, Haesebrouck F, Garmyn A, Devreese M. 2022. Dose-dependent impact of enrofloxacin on broiler chicken gut resistome is mitigated by synbiotic application. Front Microbiol 13:869538. doi:10.3389/fmicb.2022.869538 35992659 PMC9386515

[24] Fan Y, Forgie AJ, Ju T, Marcolla C, Inglis T, McMullen LM, Willing BP, Korver DR. 2022. The use of disinfectant in barn cleaning alters microbial composition and increases carriage of Campylobacter jejuni in Broiler Chickens. Appl Environ Microbiol 88:e0029522. doi:10.1128/aem.00295-22 35475671 PMC9128520

[25] Chen S, Zhou Y, Chen Y, Gu J. 2018. Fastp: an ultra-fast all-in-one FASTQ preprocessor. Bioinformatics 34:i884–i890. doi:10.1093/bioinformatics/bty560 30423086 PMC6129281

[26] Cunningham F, Allen JE, Allen J, Alvarez-Jarreta J, Amode MR, Armean IM, Austine-Orimoloye O, Azov AG, Barnes I, Bennett R, et al.. 2022. Ensembl 2022. Nucleic Acids Res 50:D988–D995. doi:10.1093/nar/gkab1049 34791404 PMC8728283

[27] Wood DE, Lu J, Langmead B. 2019. Improved metagenomic analysis with Kraken 2. Genome Biol 20:257. doi:10.1186/s13059-019-1891-0 31779668 PMC6883579

[28] Hyatt D, Chen G-L, Locascio PF, Land ML, Larimer FW, Hauser LJ. 2010. Prodigal: prokaryotic gene recognition and translation initiation site identification. BMC Bioinformatics 11:1–11. doi:10.1186/1471-2105-11-119 20211023 PMC2848648

[29] Li D, Liu C-M, Luo R, Sadakane K, Lam T-W. 2015. MEGAHIT: an ultra-fast single-node solution for large and complex metagenomics assembly via succinct de Bruijn graph. Bioinformatics 31:1674–1676. doi:10.1093/bioinformatics/btv033 25609793

[30] Caspi R, Billington R, Ferrer L, Foerster H, Fulcher CA, Keseler IM, Kothari A, Krummenacker M, Latendresse M, Mueller LA, Ong Q, Paley S, Subhraveti P, Weaver DS, Karp PD. 2016. The MetaCyc database of metabolic pathways and enzymes and the BioCyc collection of pathway/genome databases. Nucleic Acids Res 44:D471–D480. doi:10.1093/nar/gkv1164 26527732 PMC4702838

[31] Beghini F, McIver LJ, Blanco-Míguez A, Dubois L, Asnicar F, Maharjan S, Mailyan A, Manghi P, Scholz M, Thomas AM, Valles-Colomer M, Weingart G, Zhang Y, Zolfo M, Huttenhower C, Franzosa EA, Segata N. 2021. Integrating taxonomic, functional, and strain-level profiling of diverse microbial communities with bioBakery 3. eLife 10:e65088. doi:10.7554/eLife.65088 33944776 PMC8096432

[32] Alcock BP, Raphenya AR, Lau TTY, Tsang KK, Bouchard M, Edalatmand A, Huynh W, Nguyen A-LV, Cheng AA, Liu S, et al.. 2020. CARD 2020: antibiotic resistome surveillance with the comprehensive antibiotic resistance database. Nucleic Acids Res 48:D517–D525. doi:10.1093/nar/gkz935 31665441 PMC7145624

[33] Love MI, Huber W, Anders S. 2014. Moderated estimation of fold change and dispersion for RNA-seq data with DESeq2. Genome Biol 15:550. doi:10.1186/s13059-014-0550-8 25516281 PMC4302049

[34] Laukens D, Brinkman BM, Raes J, De Vos M, Vandenabeele P. 2016. Heterogeneity of the gut microbiome in mice: guidelines for optimizing experimental design. FEMS Microbiol Rev 40:117–132. doi:10.1093/femsre/fuv036 26323480 PMC4703068

[35] Kollarcikova M, Kubasova T, Karasova D, Crhanova M, Cejkova D, Sisak F, Rychlik I. 2019. Use of 16S rRNA gene sequencing for prediction of new opportunistic pathogens in chicken ileal and cecal microbiota. Poult Sci 98:2347–2353. doi:10.3382/ps/pey594 30624758

[36] Ceelen L, Decostere A, Verschraegen G, Ducatelle R, Haesebrouck F. 2005. Prevalence of Helicobacter pullorum among patients with gastrointestinal disease and clinically healthy persons. J Clin Microbiol 43:2984–2986. doi:10.1128/JCM.43.6.2984-2986.2005 15956438 PMC1151964

[37] Rocha M, Avenaud P, Ménard A, Le Bail B, Balabaud C, Bioulac-Sage P, de Magalhães Queiroz DM, Mégraud F. 2005. Association of Helicobacter species with hepatitis C cirrhosis with or without Hepatocellular carcinoma. Gut 54:396–401. doi:10.1136/gut.2004.042168 15710989 PMC1774397

[38] Wang L, Lilburn M, Yu Z. 2016. Intestinal microbiota of broiler chickens as affected by litter management regimens. Front Microbiol 7:593. doi:10.3389/fmicb.2016.00593 27242676 PMC4870231

[39] Lund M, Bjerrum L, Pedersen K. 2010. Quantification of Faecalibacterium prausnitzii- and Subdoligranulum variabile-like bacteria in the cecum of chickens by real-time PCR. Poult Sci 89:1217–1224. doi:10.3382/ps.2010-00653 20460669

[40] Louis P, Flint HJ. 2009. Diversity, metabolism and microbial ecology of butyrate-producing bacteria from the human large intestine. FEMS Microbiol Lett 294:1–8. doi:10.1111/j.1574-6968.2009.01514.x 19222573

[41] Wang XJ, Feng JH, Zhang MH, Li XM, Ma DD, Chang SS. 2018. Effects of high ambient temperature on the community structure and composition of ileal microbiome of broilers. Poult Sci 97:2153–2158. doi:10.3382/ps/pey032 29562351

[42] Reichardt N, Duncan SH, Young P, Belenguer A, McWilliam Leitch C, Scott KP, Flint HJ, Louis P. 2014. Phylogenetic distribution of three pathways for propionate production within the human gut microbiota. ISME J 8:1323–1335. doi:10.1038/ismej.2014.14 24553467 PMC4030238

[43] Sakamoto M, Lan PTN, Benno Y. 2007. Barnesiella viscericola gen. nov., sp. nov., a novel member of the family Porphyromonadaceae isolated from chicken caecum. Int J Syst Evol Microbiol 57:342–346. doi:10.1099/ijs.0.64709-0 17267976

[44] Weiss GA, Chassard C, Hennet T. 2014. Selective proliferation of intestinal Barnesiella under fucosyllactose supplementation in mice. Br J Nutr 111:1602–1610. doi:10.1017/S0007114513004200 24411010

[45] Wilson KH, Ikeda JS, Blitchington RB. 1997. Phylogenetic placement of community members of human colonic biota. Clin Infect Dis 25 Suppl 2:S114–S116. doi:10.1086/516230 9310646

[46] Hauryliuk V, Atkinson GC, Murakami KS, Tenson T, Gerdes K. 2015. Recent functional insights into the role of (p)ppGpp in bacterial physiology. Nat Rev Microbiol 13:298–309. doi:10.1038/nrmicro3448 25853779 PMC4659695

[47] Schofield WB, Zimmermann-Kogadeeva M, Zimmermann M, Barry NA, Goodman AL. 2018. The stringent response determines the ability of a commensal bacterium to survive starvation and to persist in the gut. Cell Host & Microbe 24:120–132. doi:10.1016/j.chom.2018.06.002 30008292 PMC6086485

[48] Boaretti M, Lleò MM, Bonato B, Signoretto C, Canepari P. 2003. Involvement of rpoS in the survival of Escherichia coli in the viable but non-culturable state. Environ Microbiol 5:986–996. doi:10.1046/j.1462-2920.2003.00497.x 14510852

[49] Salzer A, Wolz C. 2023. Role of (p)ppGpp in antibiotic resistance, tolerance, persistence and survival in Firmicutes. Microlife 4:uqad009. doi:10.1093/femsml/uqad009 37223729 PMC10117726

[50] Stenz L, Francois P, Whiteson K, Wolz C, Linder P, Schrenzel J. 2011. The CodY pleiotropic repressor controls virulence in gram-positive pathogens. FEMS Immunol Med Microbiol 62:123–139. doi:10.1111/j.1574-695X.2011.00812.x 21539625

[51] Kaiser JC, King AN, Grigg JC, Sheldon JR, Edgell DR, Murphy MEP, Brinsmade SR, Heinrichs DE. 2018. Repression of branched-chain amino acid synthesis in Staphylococcus aureus is mediated by isoleucine via CodY, and by a leucine-rich attenuator peptide. PLoS Genet 14:e1007159. doi:10.1371/journal.pgen.1007159 29357354 PMC5794164

[52] Brinsmade SR, Kleijn RJ, Sauer U, Sonenshein AL. 2010. Regulation of CodY activity through modulation of intracellular branched-chain amino acid pools. J Bacteriol 192:6357–6368. doi:10.1128/JB.00937-10 20935095 PMC3008522

[53] Gong L, Wang B, Zhou Y, Tang L, Zeng Z, Zhang H, Li W. 2021. Protective effects of Lactobacillus plantarum 16 and Paenibacillus polymyxa 10 against Clostridium perfringens Infection in Broilers. Front Immunol 11:628374. doi:10.3389/fimmu.2020.628374 33679724 PMC7930238

[54] McNally DJ, Aubry AJ, Hui JPM, Khieu NH, Whitfield D, Ewing CP, Guerry P, Brisson J-R, Logan SM, Soo EC. 2007. Targeted metabolomics analysis of Campylobacter coli VC167 reveals legionaminic acid derivatives as novel flagellar glycans. J Biol Chem 282:14463–14475. doi:10.1074/jbc.M611027200 17371878

[55] Thibault P, Logan SM, Kelly JF, Brisson JR, Ewing CP, Trust TJ, Guerry P. 2001. Identification of the carbohydrate moieties and glycosylation motifs in Campylobacter jejuni flagellin. J Biol Chem 276:34862–34870. doi:10.1074/jbc.M104529200 11461915

[56] Schirm M, Soo EC, Aubry AJ, Austin J, Thibault P, Logan SM. 2003. Structural, genetic and functional characterization of the flagellin glycosylation process in Helicobacter pylori. Mol Microbiol 48:1579–1592. doi:10.1046/j.1365-2958.2003.03527.x 12791140

[57] Yang H, Byelashov OA, Geornaras I, Goodridge LD, Nightingale KK, Belk KE, Smith GC, Sofos JN. 2010. Presence of antibiotic-resistant commensal bacteria in samples from agricultural, city, and national park environments evaluated by standard culture and real-time PCR methods. Can J Microbiol 56:761–770. doi:10.1139/W10-060 20921986

[58] Kim SY, Kuppusamy S, Kim JH, Yoon YE, Kim KR, Lee YB. 2016. Occurrence and diversity of tetracycline resistance genes in the agricultural soils of South Korea. Environ Sci Pollut Res 23:22190–22196. doi:10.1007/s11356-016-7574-4 27638788

[59] Li C, Jiang C, Wu Z, Cheng B, An X, Wang H, Sun Y, Huang M, Chen X, Wang J. 2018. Diversity of antibiotic resistance genes and encoding ribosomal protection proteins gene in livestock waste polluted environment. Journal of Environmental Science and Health, Part B 53:423–433. doi:10.1080/03601234.2018.1438836 29469609

[60] Nogrado K, Unno T, Hur H-G, Lee J-H. 2021. Tetracycline-resistant bacteria and ribosomal protection protein genes in soils from selected agricultural fields and livestock farms. Appl Biol Chem 64:1–9. doi:10.1186/s13765-021-00613-6

[61] Luiken RE, Heederik DJ, Scherpenisse P, Van Gompel L, van Heijnsbergen E, Greve GD, Jongerius-Gortemaker BG, Tersteeg-Zijderveld MH, Fischer J, Juraschek K, Skarżyńska M, Zając M, Wasyl D, Wagenaar JA, Smit LA, Wouters IM, Mevius DJ, Schmitt H, EFFORT-group. 2022. Determinants for antimicrobial resistance genes in farm dust on 333 poultry and pig farms in nine European countries. Environ Res 208:112715. doi:10.1016/j.envres.2022.112715 35033551

[62] Juricova H, Matiasovicova J, Kubasova T, Cejkova D, Rychlik I. 2021. The distribution of antibiotic resistance genes in chicken gut microbiota commensals. Sci Rep 11:3290. doi:10.1038/s41598-021-82640-3 33558560 PMC7870933

[63] Wang Y, Hu Y, Cao J, Bi Y, Lv N, Liu F, Liang S, Shi Y, Jiao X, Gao GF, Zhu B. 2019. Antibiotic resistance gene reservoir in live poultry markets. J Infect 78:445–453. doi:10.1016/j.jinf.2019.03.012 30935879

[64] Chicken Farmers of Canada. 2018. AMU strategy - a prescription for change

[65] Xu J, Sangthong R, McNeil E, Tang R, Chongsuvivatwong V. 2020. Antibiotic use in chicken farms in northwestern China. Antimicrob Resist Infect Control 9:1–9. doi:10.1186/s13756-019-0672-6 31921416 PMC6947973

[66] Noyes NR, Yang X, Linke LM, Magnuson RJ, Cook SR, Zaheer R, Yang H, Woerner DR, Geornaras I, McArt JA, Gow SP, Ruiz J, Jones KL, Boucher CA, McAllister TA, Belk KE, Morley PS. 2016. Characterization of the resistome in manure, soil and wastewater from dairy and beef production systems. Sci Rep 6:24645. doi:10.1038/srep24645 27095377 PMC4837390

[67] Gupta CL, Blum SE, Kattusamy K, Daniel T, Druyan S, Shapira R, Krifucks O, Zhu YG, Zhou XY, Su JQ, Cytryn E. 2021. Longitudinal study on the effects of growth-promoting and therapeutic antibiotics on the dynamics of chicken cloacal and litter microbiomes and resistomes. Microbiome 9:178. doi:10.1186/s40168-021-01136-4 34454634 PMC8403378

[68] Nandi S, Maurer JJ, Hofacre C, Summers AO. 2004. Gram-positive bacteria are a major reservoir of Class 1 antibiotic resistance integrons in poultry litter. Proc Natl Acad Sci USA 101:7118–7122. doi:10.1073/pnas.0306466101 15107498 PMC406475

[69] Furtula V, Farrell EG, Diarrassouba F, Rempel H, Pritchard J, Diarra MS. 2010. Veterinary pharmaceuticals and antibiotic resistance of Escherichia coli isolates in poultry litter from commercial farms and controlled feeding trials. Poult Sci 89:180–188. doi:10.3382/ps.2009-00198 20008817

[70] Cheng W, Chen H, Su C, Yan S. 2013. Abundance and persistence of antibiotic resistance genes in livestock farms: a comprehensive investigation in eastern China. Environ Int 61:1–7. doi:10.1016/j.envint.2013.08.023 24091253

[71] Toh SM, Xiong L, Arias CA, Villegas MV, Lolans K, Quinn J, Mankin AS. 2007. Acquisition of a natural resistance gene renders a clinical strain of methicillin-resistant Staphylococcus aureus resistant to the synthetic antibiotic linezolid. Mol Microbiol 64:1506–1514. doi:10.1111/j.1365-2958.2007.05744.x 17555436 PMC2711439

[72] Wang M, Liu P, Xiong W, Zhou Q, Wangxiao J, Zeng Z, Sun Y. 2018. Fate of potential indicator antimicrobial resistance genes (ARGs) and bacterial community diversity in simulated manure-soil microcosms. Ecotoxicol Environ Saf 147:817–823. doi:10.1016/j.ecoenv.2017.09.055 28958128

[73] You Y, Hilpert M, Ward MJ. 2012. Detection of a common and persistent tet(L)-carrying plasmid in chicken-waste-impacted farm soil. Appl Environ Microbiol 78:3203–3213. doi:10.1128/AEM.07763-11 22389375 PMC3346469

[74] Li B, Chen D, Lin F, Wu C, Cao L, Chen H, Hu Y, Yin Y. 2022. Genomic Island-mediated horizontal transfer of the erythromycin resistance gene erm(X) among Bifidobacteria. Appl Environ Microbiol 88:e0041022. doi:10.1128/aem.00410-22 35477272 PMC9128502

[75] Shoemaker NB, Vlamakis H, Hayes K, Salyers AA. 2001. Evidence for extensive resistance gene transfer among Bacteroides spp. and among Bacteroides and other genera in the human colon. Appl Environ Microbiol 67:561–568. doi:10.1128/AEM.67.2.561-568.2001 11157217 PMC92621

[76] Chen L, Huang J, Huang X, He Y, Sun J, Dai X, Wang X, Shafiq M, Wang L. 2021. Horizontal transfer of different erm(B)-carrying mobile elements among Streptococcus suis strains with different serotypes. Front Microbiol 12:628740. doi:10.3389/fmicb.2021.628740 33841355 PMC8032901

[77] Park AK, Kim H, Jin HJ. 2010. Phylogenetic analysis of rRNA methyltransferases, Erm and KsgA, as related to antibiotic resistance. FEMS Microbiol Lett 309:151–162. doi:10.1111/j.1574-6968.2010.02031.x 20618865

[78] Sjlund M, Wreiber K, Andersson DI, Blaser MJ, Engstrand L. 2003. Long-term persistence of resistant Enterococcus species after antibiotics to eradicate Helicobacter pylori. Ann Intern Med 139:483–487. doi:10.7326/0003-4819-139-6-200309160-00011 13679325

[79] Just NA, Létourneau V, Kirychuk SP, Singh B, Duchaine C. 2012. Potentially pathogenic bacteria and antimicrobial resistance in bioaerosols from cage-housed and floor-housed poultry operations. Ann Occup Hyg 56:440–449. doi:10.1093/annhyg/mer105 22156572

[80] Wada Y, Irekeola AA, Shueb RH, Wada M, Afolabi HA, Yean CY, Harun A, Zaidah AR. 2022. Prevalence of vancomycin-resistant Enterococcus (VRE) in poultry in Malaysia: the first meta-analysis and systematic review. Antibiotics (Basel) 11:171. doi:10.3390/antibiotics11020171 35203775 PMC8868266

[81] Leinweber H, Alotaibi SMI, Overballe-Petersen S, Hansen F, Hasman H, Bortolaia V, Hammerum AM, Ingmer H. 2018. Vancomycin resistance in Enterococcus faecium isolated from Danish chicken meat is located on a pVEF4-like plasmid persisting in poultry for 18 years. Int J Antimicrob Agents 52:283–286. doi:10.1016/j.ijantimicag.2018.03.019 29621590

[82] Birkegård AC, Græsbøll K, Clasen J, Halasa T, Toft N, Folkesson A. 2019. Continuing occurrence of vancomycin resistance determinants in Danish pig farms 20 years after removing exposure to avoparcin. Vet Microbiol 232:84–88. doi:10.1016/j.vetmic.2019.04.007 31030850

[83] Dahl KH, Mater DDG, Flores MJ, Johnsen PJ, Midtvedt T, Corthier G, Sundsfjord A. 2007. Transfer of plasmid and chromosomal glycopeptide resistance determinants occurs more readily in the digestive tract of mice than in vitro and exconjugants can persist stably in vivo in the absence of glycopeptide selection. J Antimicrob Chemother 59:478–486. doi:10.1093/jac/dkl530 17283034

[84] Lebeaux RM, Coker MO, Dade EF, Palys TJ, Morrison HG, Ross BD, Baker ER, Karagas MR, Madan JC, Hoen AG. 2021. The infant gut resistome is associated with E. coli and early-life exposures. BMC Microbiol 21:201. doi:10.1186/s12866-021-02129-x 34215179 PMC8252198

[85] Weston N, Sharma P, Ricci V, Piddock LJV. 2018. Regulation of the AcrAB-TolC efflux pump in Enterobacteriaceae. Res Microbiol 169:425–431. doi:10.1016/j.resmic.2017.10.005 29128373

[86] Slipski CJ, Zhanel GG, Bay DC. 2018. Biocide selective TolC-independent Efflux pumps in Enterobacteriaceae. J Membrane Biol 251:15–33. doi:10.1007/s00232-017-9992-8 29063140 PMC5840245

[87] Achard A, Villers C, Pichereau V, Leclercq R. 2005. New lnu (C) gene conferring resistance to lincomycin by nucleotidylation in Streptococcus agalactiae UCN36. Antimicrob Agents Chemother (Bethesda) 49:2716–2719. doi:10.1128/AAC.49.7.2716-2719.2005 PMC1168647 15980341

[88] Kim Y, Leung MHY, Kwok W, Fournié G, Li J, Lee PKH, Pfeiffer DU. 2020. Antibiotic resistance gene sharing networks and the effect of dietary nutritional content on the canine and feline gut resistome. Anim Microbiome 2:4. doi:10.1186/s42523-020-0022-2 33500005 PMC7807453

[89] Hull DM, Harrell E, van Vliet AHM, Correa M, Thakur S. 2021. Antimicrobial resistance and interspecies gene transfer in Campylobacter coli and Campylobacter jejuni isolated from food animals, poultry processing, and retail meat in North Carolina, 2018-2019. PLoS One 16:e0246571. doi:10.1371/journal.pone.0246571 33571292 PMC7877606

[90] Mourkas E, Florez-Cuadrado D, Pascoe B, Calland JK, Bayliss SC, Mageiros L, Méric G, Hitchings MD, Quesada A, Porrero C, Ugarte-Ruiz M, Gutiérrez-Fernández J, Domínguez L, Sheppard SK. 2019. Gene pool transmission of multidrug resistance among Campylobacter from livestock, sewage and human disease. Environ Microbiol 21:4597–4613. doi:10.1111/1462-2920.14760 31385413 PMC6916351

[91] Zhao S, Tyson GH, Chen Y, Li C, Mukherjee S, Young S, Lam C, Folster JP, Whichard JM, McDermott PF. 2016. Whole-genome sequencing analysis accurately predicts antimicrobial resistance phenotypes in Campylobacter spp. Appl Environ Microbiol 82:459–466. doi:10.1128/AEM.02873-15 26519386 PMC4711122

[92] Tang Y, Jiang Q, Tang H, Wang Z, Yin Y, Ren F, Kong L, Jiao X, Huang J. 2020. Characterization and prevalence of Campylobacter spp. from broiler chicken rearing period to the slaughtering process in Eastern China. Front Vet Sci 7:227. doi:10.3389/fvets.2020.00227 32426383 PMC7203416

[93] Nijland R, Burgess JG, Errington J, Veening J-W. 2010. Transformation of environmental Bacillus subtilis isolates by transiently inducing genetic competence. PLoS One 5:e9724. doi:10.1371/journal.pone.0009724 20300532 PMC2838798

[94] Forsberg KJ, Patel S, Gibson MK, Lauber CL, Knight R, Fierer N, Dantas G. 2014. Bacterial phylogeny structures soil resistomes across habitats. Nature 509:612–616. doi:10.1038/nature13377 24847883 PMC4079543

