# Supplementary material for: Chicken cecal microbial functional gene content and resistome differ by age and barn disinfection practice
Source: Microbiol Spectr. 2025 Dec 26;14(2):e03737-25. doi: 10.1128/spectrum.03737-25 (PMC12889059; doi:10.1128/spectrum.03737-25)
Supplement: Supplemental material — Fig. S1 to S3; Table S1. [file spectrum.03737-25-s0001.docx]

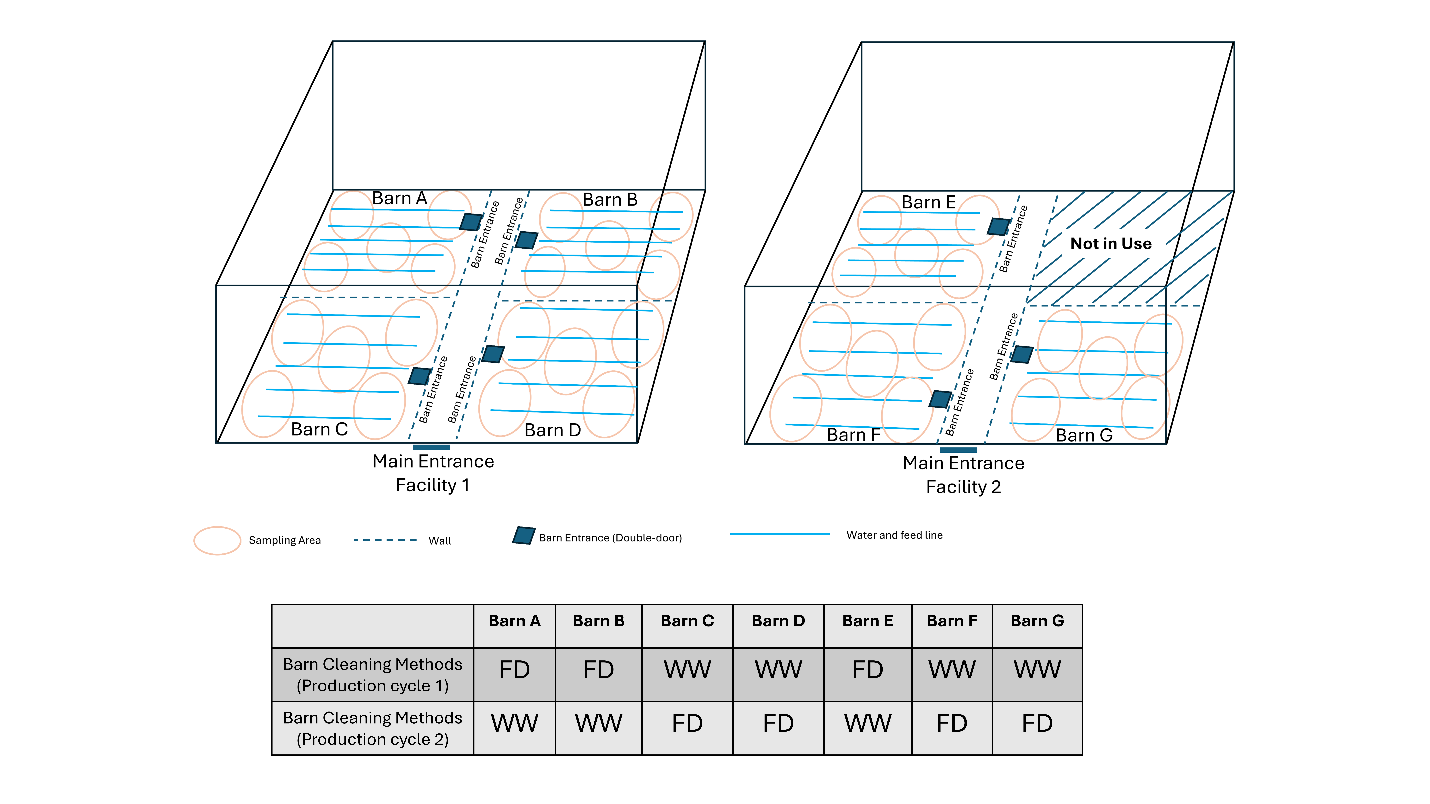


**Figure S1** Barns and treatment allocation.

Graph shows barns and treatment allocation. FD, Full Disinfection, WW, Water-wash


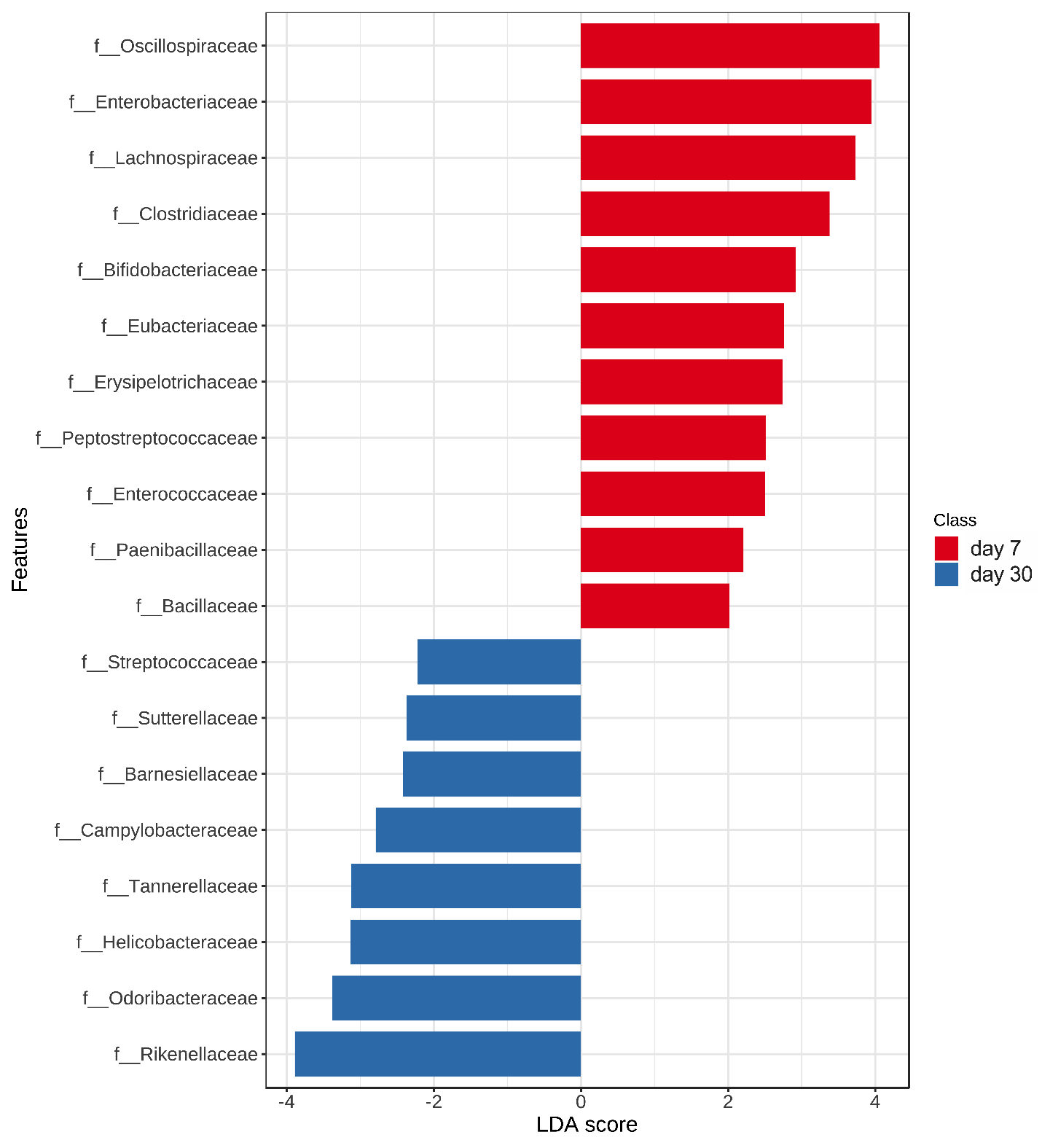


**Figure S2** LEfSe results of differentially abundant bacterial taxa on the family level between the cecal microbiota of day 7 and day 30 chickens.

Result suggested 11 and 8 bacteria families associated with D7 and D30 chicken cecal microbiota, respectively. f__, family.

**Figure S3** Spearman correlation between the relative abundance of bacterial taxa (species level) and ARG families.

Graph shows bacterial species that were correlated with antibiotic resistance gene families. Notably, among all bacterial species in the chicken gut microbiota, *Escherichia coli* was positively correlated with detected families of antibiotic resistant genes. s__, species; *, FDR P < 0.05; **, FDR P < 0.01.

**Table S1**: Genetic pathways harbored by the cecal microbial communities associated with chicken age. Negative log2 fold-change and positive log2 fold-change values indicate associations with D7 and D30 microbial functional capacities, respectively.

| Age-related differentiate abundant pathways | log2 fold-change | *P*-value | FDR *P* value |
| --- | --- | --- | --- |
| GALACTARDEG-PWY: D-galactarate degradation I | -2.2687 | 1.77E-28 | 3.07E-27 |
| GLUCARGALACTSUPER-PWY: super-pathway of D-glucarate and D-galactarate degradation | -2.2687 | 1.77E-28 | 3.07E-27 |
| PWY0-1477: ethanolamine utilization | -2.2306 | 7.78E-57 | 2.43E-54 |
| PWY-6588: pyruvate fermentation to acetone | -2.227 | 3.61E-33 | 1.03E-31 |
| PWY66-409: super-pathway of purine nucleotide salvage | -2.0384 | 1.78E-22 | 2.22E-21 |
| GLUCARDEG-PWY: D-glucarate degradation I | -2.0347 | 2.28E-28 | 3.74E-27 |
| PWY-6961: L-ascorbate degradation II (bacterial, aerobic) | -1.9453 | 1.30E-19 | 1.45E-18 |
| PWY0-301: L-ascorbate degradation I (bacterial, anaerobic) | -1.833 | 9.25E-18 | 8.74E-17 |
| PWY0-1297: super-pathway of purine deoxyribonucleosides degradation | -1.7944 | 1.41E-29 | 3.14E-28 |
| P161-PWY: acetylene degradation (anaerobic) | -1.792 | 5.50E-39 | 3.43E-37 |
| GALACTITOLCAT-PWY: galactitol degradation | -1.7755 | 3.57E-20 | 4.28E-19 |
| PWY0-1277: 3-phenylpropanoate and 3-(3-hydroxyphenyl) propanoate degradation | -1.7474 | 4.10E-16 | 3.12E-15 |
| FAO-PWY: fatty acid &beta;-oxidation I (generic) | -1.7348 | 3.13E-14 | 1.88E-13 |
| PWY-7013: (S)-propane-1,2-diol degradation | -1.709 | 1.40E-10 | 6.31E-10 |
| PWY-5723: Rubisco shunt | -1.7071 | 2.08E-14 | 1.27E-13 |
| SALVADEHYPOX-PWY: adenosine nucleotides degradation II | -1.7038 | 6.61E-34 | 2.58E-32 |
| HCAMHPDEG-PWY: 3-phenylpropanoate and 3-(3-hydroxyphenyl) propanoate degradation to 2-hydroxypentadienoate | -1.6991 | 3.57E-16 | 2.86E-15 |
| PWY-6690: cinnamate and 3-hydroxycinnamate degradation to 2-hydroxypentadienoate | -1.6991 | 3.69E-16 | 2.88E-15 |
| PRPP-PWY: super-pathway of histidine, purine, and pyrimidine biosynthesis | -1.6672 | 5.68E-10 | 2.33E-09 |
| AST-PWY: L-arginine degradation II (AST pathway) | -1.6357 | 1.97E-16 | 1.62E-15 |
| PWY-5676: acetyl-CoA fermentation to butanoate II | -1.6081 | 4.02E-40 | 3.13E-38 |
| PWY-6606: guanosine nucleotides degradation II | -1.6023 | 8.56E-29 | 1.67E-27 |
| PWY-5136: fatty acid &beta;-oxidation II (plant peroxisome) | -1.5914 | 1.14E-25 | 1.61E-24 |
| PWY0-1338: polymyxin resistance | -1.5642 | 1.21E-14 | 8.05E-14 |
| PWY-7242: D-fructuronate degradation | -1.56 | 9.25E-23 | 1.20E-21 |
| PWY-7858: (5Z)-dodecenoate biosynthesis II | -1.5501 | 3.57E-13 | 1.95E-12 |
| PWY0-1298: super-pathway of pyrimidine deoxyribonucleosides degradation | -1.5497 | 1.58E-23 | 2.14E-22 |
| ORNDEG-PWY: super-pathway of ornithine degradation | -1.546 | 7.28E-13 | 3.92E-12 |
| PWY-5138: fatty acid &beta;-oxidation IV (unsaturated, even number) | -1.5453 | 8.38E-16 | 6.22E-15 |
| PWY-5367: petroselinate biosynthesis | -1.4997 | 6.20E-15 | 4.20E-14 |
| HEXITOLDEGSUPER-PWY: super-pathway of hexitol degradation (bacteria) | -1.4995 | 4.86E-14 | 2.81E-13 |
| PWY-7942: 5-oxo-L-proline metabolism | -1.4813 | 8.78E-13 | 4.64E-12 |
| PWY4LZ-257: super-pathway of fermentation (Chlamydomonas reinhardtii) | -1.4774 | 1.09E-33 | 3.79E-32 |
| PWY-5675: nitrate reduction V (assimilatory) | -1.4423 | 7.83E-10 | 3.13E-09 |
| PWY-6507: 4-deoxy-L-threo-hex-4-enopyranuronate degradation | -1.4276 | 2.23E-17 | 2.05E-16 |
| PWY-6608: guanosine nucleotides degradation III | -1.4213 | 3.07E-28 | 4.79E-27 |
| PWY-7111: pyruvate fermentation to isobutanol (engineered) | -1.3935 | 5.58E-35 | 2.90E-33 |
| PWY0-42: 2-methylcitrate cycle I | -1.3784 | 2.57E-11 | 1.23E-10 |
| PWY0-1415: super-pathway of heme b biosynthesis from uroporphyrinogen-III | -1.372 | 8.12E-11 | 3.72E-10 |
| PWY-6353: purine nucleotides degradation II (aerobic) | -1.3545 | 2.71E-27 | 4.02E-26 |
| ECASYN-PWY: enterobacterial common antigen biosynthesis | -1.353 | 3.50E-14 | 2.06E-13 |
| PWY-6284: super-pathway of unsaturated fatty acids biosynthesis (E. coli) | -1.3379 | 2.01E-09 | 7.74E-09 |
| PWY-5860: super-pathway of demethylmenaquinol-6 biosynthesis I | -1.3299 | 4.62E-09 | 1.70E-08 |
| PWY-5100: pyruvate fermentation to acetate and lactate II | -1.3127 | 2.39E-17 | 2.13E-16 |
| PWY-6936: seleno-amino acid biosynthesis (plants) | -1.3024 | 3.64E-33 | 1.03E-31 |
| PWY-7118: chitin deacetylation | -1.291 | 1.76E-09 | 6.87E-09 |
| PWY-I9: L-cysteine biosynthesis VI (from L-methionine) | -1.289 | 1.15E-18 | 1.16E-17 |
| PWY-7237: myo-, chiro- and scyllo-inositol degradation | -1.2788 | 4.93E-19 | 5.31E-18 |
| PWY-5850: super-pathway of menaquinol-6 biosynthesis | -1.2775 | 2.13E-07 | 6.21E-07 |
| PWY0-781: aspartate super-pathway | -1.2458 | 4.30E-09 | 1.60E-08 |
| P41-PWY: pyruvate fermentation to acetate and (S)-lactate I | -1.2414 | 2.77E-18 | 2.70E-17 |
| P4-PWY: super-pathway of L-lysine, L-threonine and L-methionine biosynthesis I | -1.2388 | 5.20E-09 | 1.84E-08 |
| PWY-5384: sucrose degradation IV (sucrose phosphorylase) | -1.2329 | 1.08E-11 | 5.33E-11 |
| GLYCOLYSIS-TCA-GLYOX-BYPASS: super-pathway of glycolysis, pyruvate dehydrogenase, TCA, and glyoxylate bypass | -1.2268 | 3.14E-08 | 1.01E-07 |
| GLYOXYLATE-BYPASS: glyoxylate cycle | -1.2197 | 5.20E-10 | 2.18E-09 |
| HEME-BIOSYNTHESIS-II-1: heme b biosynthesis V (aerobic) | -1.218 | 4.96E-09 | 1.79E-08 |
| KETOGLUCONMET-PWY: ketogluconate metabolism | -1.2137 | 4.41E-08 | 1.39E-07 |
| PWY-6285: super-pathway of fatty acids biosynthesis (E. coli) | -1.14 | 7.36E-06 | 1.74E-05 |
| PWY-561: super-pathway of glyoxylate cycle and fatty acid degradation | -1.1355 | 1.40E-07 | 4.24E-07 |
| TCA-GLYOX-BYPASS: super-pathway of glyoxylate bypass and TCA | -1.0869 | 4.48E-07 | 1.24E-06 |
| PWY-7385: 1,3-propanediol biosynthesis (engineered) | -1.0835 | 2.85E-07 | 8.17E-07 |
| PWY-5920: super-pathway of heme b biosynthesis from glycine | -1.062 | 1.85E-08 | 6.19E-08 |
| PWY-8004: Entner-Doudoroff pathway I | -1.0498 | 1.65E-13 | 9.18E-13 |
| PWY-7211: super-pathway of pyrimidine deoxyribonucleotides de novo biosynthesis | -1.0478 | 3.45E-12 | 1.79E-11 |
| HOMOSER-METSYN-PWY: L-methionine biosynthesis I | -1.0376 | 1.64E-07 | 4.88E-07 |
| P108-PWY: pyruvate fermentation to propanoate I | -1.0221 | 1.14E-05 | 2.59E-05 |
| PWY66-389: phytol degradation | -1.0002 | 3.90E-05 | 8.51E-05 |
| PPGPPMET-PWY: ppGpp metabolism | 1.1234 | 4.56E-06 | 1.12E-05 |
| PWY-8073: lipid IVA biosynthesis (P. putida) | 1.1533 | 1.32E-14 | 8.55E-14 |
| NAGLIPASYN-PWY: lipid IVA biosynthesis (E. coli) | 1.1533 | 1.34E-14 | 8.55E-14 |
| P42-PWY: incomplete reductive TCA cycle | 1.1623 | 7.09E-10 | 2.87E-09 |
| PWY-1269: CMP-3-deoxy-D-manno-octulosonate biosynthesis | 1.1729 | 1.60E-16 | 1.35E-15 |
| PWY-5121: super-pathway of geranylgeranyl diphosphate biosynthesis II (via MEP) | 1.2348 | 5.25E-10 | 2.18E-09 |
| PWY-6969: TCA cycle V (2-oxoglutarate synthase) | 1.3108 | 3.84E-15 | 2.66E-14 |
| PWY-6902: chitin degradation II (Vibrio) | 1.3415 | 4.09E-08 | 1.30E-07 |
| PWY-7388: octanoyl-[acyl-carrier protein] biosynthesis (mitochondria, yeast) | 1.4459 | 5.00E-09 | 1.79E-08 |
| CITRULBIO-PWY: L-citrulline biosynthesis | 1.4899 | 9.41E-19 | 9.79E-18 |
| PWY-7254: TCA cycle VII (acetate-producers) | 1.579 | 1.43E-14 | 8.92E-14 |
| PWY0-1241: ADP-L-glycero-&beta;-D-manno-heptose biosynthesis | 1.6134 | 6.43E-12 | 3.24E-11 |
| PWY-6834: spermidine biosynthesis III | 1.6637 | 6.04E-09 | 2.12E-08 |
| PWY-7371: 1,4-dihydroxy-6-naphthoate biosynthesis II | 2.4597 | 5.28E-12 | 2.70E-11 |
| PWY-7392: taxadiene biosynthesis (engineered) | 2.4911 | 1.14E-30 | 2.97E-29 |
| PWY-6922: L-N & delta; -acetylornithine biosynthesis | 2.5988 | 2.77E-29 | 5.77E-28 |
| PWY-4984: urea cycle | 2.6001 | 4.86E-17 | 4.21E-16 |
| PWY-7992: super-pathway of menaquinol-8 biosynthesis III | 2.7342 | 5.23E-14 | 2.97E-13 |
| PWY-5030: L-histidine degradation III | 2.9661 | 7.08E-30 | 1.70E-28 |
| POLYAMINSYN3-PWY: super-pathway of polyamine biosynthesis II | 2.9832 | 8.84E-49 | 9.19E-47 |
| PWY-5005: biotin biosynthesis II | 3.8625 | 2.64E-34 | 1.18E-32 |
| PWY-6906: chitin derivatives degradation | 4.0059 | 1.29E-49 | 2.01E-47 |
